# A hybrid deep learning-based approach for optimal genotype by environment selection

**DOI:** 10.3389/frai.2024.1312115

**Published:** 2024-12-11

**Authors:** Zahra Khalilzadeh, Motahareh Kashanian, Saeed Khaki, Lizhi Wang

**Affiliations:** ^1^Department of Industrial and Manufacturing Systems Engineering, Iowa State University, Ames, IA, United States; ^2^School of Industrial Engineering and Management, Oklahoma State University, Stillwater, OK, United States

**Keywords:** convolutional neural network, genotype selection, crop yield prediction, Generalized Ensemble Method, genotype-environment interaction, feature importance analysis

## Abstract

The ability to accurately predict the yields of different crop genotypes in response to weather variability is crucial for developing climate resilient crop cultivars. Genotype-environment interactions introduce large variations in crop-climate responses, and are hard to factor in to breeding programs. Data-driven approaches, particularly those based on machine learning, can help guide breeding efforts by factoring in genotype-environment interactions when making yield predictions. Using a new yield dataset containing 93,028 records of soybean hybrids across 159 locations, 28 states, and 13 years, with 5,838 distinct genotypes and daily weather data over a 214-day growing season, we developed two convolutional neural network (CNN) models: one that integrates CNN and fully-connected neural networks (CNN model), and another that incorporates a long short-term memory (LSTM) layer after the CNN component (CNN-LSTM model). By applying the Generalized Ensemble Method (GEM), we combined the CNN-based models and optimized their weights to improve overall predictive performance. The dataset provided unique genotype information on seeds, enabling an investigation into the potential of planting different genotypes based on weather variables. We employed the proposed GEM model to identify the best-performing genotypes across various locations and weather conditions, making yield predictions for all potential genotypes in each specific setting. To assess the performance of the GEM model, we evaluated it on unseen genotype-location combinations, simulating real-world scenarios where new genotypes are introduced. By combining the base models, the GEM ensemble approach provided much better prediction accuracy compared to using the CNN-LSTM model alone and slightly better accuracy than the CNN model, as measured by both RMSE and MAE on the validation and test sets. The proposed data-driven approach can be valuable for genotype selection in scenarios with limited testing years. In addition, we explored the impact of incorporating state-level soil data alongside the weather, location, genotype and year variables. Due to data constraints, including the absence of latitude and longitude details, we used uniform soil variables for all locations within the same state. This limitation restricted our spatial information to state-level knowledge. Our findings suggested that integrating state-level soil variables did not substantially enhance the predictive capabilities of the models. We also performed a feature importance analysis using RMSE change to identify crucial predictors. Location showed the highest RMSE change, followed by genotype and year. Among weather variables, maximum direct normal irradiance (MDNI) and average precipitation (AP) displayed higher RMSE changes, indicating their importance.

## 1 Introduction

The world's population is projected to reach almost 10 billion by 2050 (Nations et al., 2017), and climate change is expected to have a significant impact on crop yields in the coming years. As a result, there is an urgent need to increase crop production in order to feed the growing population. The current global food production systems are facing several challenges such as the increasing frequency and severity of droughts, floods, heatwaves and increased pests and diseases, which are all associated with climate change (Kumar, 2016). These challenges are likely to affect crop yields and food security, making it essential to develop new strategies to increase crop production.

One of the main strategies for increasing crop production is to develop climate-resilient crops through breeding programs (Hafeez et al., 2023). This involves selecting and crossbreeding plants that are better able to withstand the effects of climate change, such as drought or heat stress. Despite the focus on climate resilience in breeding programs, there is mounting evidence of the difficulties and challenges in creating crops capable of handling the effects of climate change. These challenges stem from the contradiction between the pressing need for breeding in response to climate change and the inadequate understanding of how genotype and environment interact with each other (Xiong et al., 2022). Another approach is to use crop simulation models that integrate environmental information and tools into the breeding analysis process to tackle the effects of climate change and anticipate crop growth and yield under different climate scenarios (de Los Campos et al., 2020; Heslot et al., 2014). However, crop simulation models have limitations, particularly related to their complexity and the challenge of fully capturing all interactions of multiple factors such as genetics, environment, and management practices. While these models are designed to simulate many of these interactions, the primary issue often lies in the availability and quality of input data required for accurate calibration and prediction (Lobell and Burke, 2010). Additionally, computational resources, the limitation of analyzing a limited number of genotypes, and simplification of reality in the models are other limitations of simulation crop modeling (Roberts et al., 2017; Hajjarpoor et al., 2022). To overcome the limitations of crop growth models, studies are emerging recently to utilize statistical methods as promising alternatives and complementary tools. Among these methods, Machine Learning (ML) is a practical statistical approach that has gained popularity due to advancements in big-data technologies and high-performance computing. ML algorithms can help farmers to increase crop production in response to climate change by providing capabilities such as crop yield prediction (Shahhosseini et al., 2021; Khaki and Wang, 2019), climate change impact modeling (Crane-Droesch, 2018), climate-smart crop breeding (Xu et al., 2022), automation of farming equipment (Patil and Thorat, 2016), market price prediction (Chen et al., 2021), water management optimization (Lowe et al., 2022), disease and pest forecasting (Domingues et al., 2022), and precision agriculture (Sharma et al., 2020). These capabilities can help farmers to plan for and adapt to changing weather patterns, identify resilient crops, optimize crop management practices, and make better decisions to increase crop production. The challenge of effectively training ML algorithms is posed by the inconsistent spatial and temporal data regarding some of the production and management inputs, such as planting date, fertilizer application rate, and crop-specific data (Srivastava et al., 2022). This is a problem that needs to be addressed for efficient ML algorithm training.

Genotype by environment interaction is a challenging factor that limits the genotype selection for increased crop yields in unseen and new environments especially with the presence of global climate change. Plant breeders typically choose hybrids based on their desired traits and characteristics, such as yield, disease resistance, and quality. They first select parent plants with desirable traits and cross them to create a new hybrid. The new hybrids are then tested in various environments to determine their performance, finally the hybrids with the highest yield are selected (Bertan et al., 2007). However, this approach can be extremely time-consuming and tedious due to the vast number of possible parent combinations that require testing (Khaki et al., 2020a). This highlights the importance of having a data driven approach to select genotypes with the highest performance in response to climates as well as other environmental variables using limited years of field testing per genotype. For example, Arzanipour and Olafsson (2022), suggests employing imputation methods to address the issue of incomplete data, particularly when certain crop types are not cultivated in every observed environment. This perspective views these absent data points not merely as traditional missing values but as potential opportunities for additional observations. In this study, we introduce a new deep learning framework for predicting crop yields using environmental data and genotype information. The framework is designed to identify the most efficient genotype for each location and environment, by first forecasting crop yields based on the given weather conditions in each location for all available genotypes, and then selecting the optimal genotype with the highest yield in each specific location and environmental scenario. This strategy helps in enhancing policy and agricultural decision-making, optimizing production, and guaranteeing food security. To the best of our knowledge this is the first study to use a deep learning approach for optimal genotype by environment selection.

Over the years, several machine learning algorithms have been employed for predicting performance of crops under different environmental conditions. These include Convolutional Neural Network (CNN; Srivastava et al., 2022), Long Short Term Memory (LSTM) networks (Shook et al., 2021), Regression Tree (RT) (Veenadhari et al., 2011), Random Forest (RF), Support Vector Machine (SVM), K-Nearest Neighbor (KNN), Extreme Gradient Boosting (XGBoost), Least Absolute Shrinkage and Selection Operator (LASSO) Regression (Kang et al., 2020), and Deep Neural Network (DNN; Khaki and Wang, 2019). In time series prediction tasks, deep neural networks have proven to be robust to inputs with noise and possess the ability to model complex non-linear functions (Dorffner, 1996). By utilizing deep learning models, it becomes possible to tackle complex data, as these models can effectively learn the non-linear relationships between the multivariate input data, which includes weather variables, maturity group/cluster information, genotype information, and the predicted yield.

Our proposed CNN-LSTM model consists of CNNs and LSTM. CNNs can handle data in multiple array formats, such as one-dimensional data like signals and sequences, two-dimensional data such as images, and three-dimensional data like videos. A typical CNN model consists of a series of convolutional and pooling layers, followed by a few fully connected layers. There are several design parameters that can be adjusted in CNNs, including the number of filters, filter size, type of padding, and stride. Filters are weight matrices used to process the input data during convolution. Padding involves adding zeroes to the input data to maintain its dimensional structure, while the stride refers to the distance by which the filter is moved during processing (Albawi et al., 2017). Recurrent Neural Networks (RNNs) are a type of deep learning model designed for handling sequential data. The key advantage of RNNs is their ability to capture time dependencies in sequential data due to their memory mechanism, allowing them to use information from previous time steps in future predictions (Sherstinsky, 2020; Lipton et al., 2015). LSTM networks are a specialized type of RNNs that address the issue of vanishing gradients in traditional RNNs (Hochreiter and Schmidhuber, 1997; Sherstinsky, 2020). LSTMs are particularly beneficial for capturing long-term dependencies in sequential data, and they maintain information for longer periods of time compared to traditional RNNs (Hochreiter and Schmidhuber, 1996). These characteristics make LSTMs highly effective for handling data with complex temporal structures, such as speech and video (Xie et al., 2019; Li et al., 2019). Furthermore, LSTMs have been successfully utilized in multivariate time series prediction problems (Shook et al., 2021; Sun et al., 2019; Gangopadhyay et al., 2018), and they are flexible and handle varying length inputs, making them suitable for processing sequential data with different lengths (Sutskever et al., 2014).

Crop yield prediction has been more recently improved by the application of deep learning methods. Khaki and Wang (2019) utilized deep neural networks to predict corn yield for various maize hybrids using environmental data and genotype information. Their study involved designing a deep neural network model that could forecast corn yield across 2,247 locations from 2008 to 2016. With regards to the accuracy of their predictions, the model they developed outperformed others such as LASSO Regression, shallow neural networks, and Regression Trees, exhibiting a Root Mean Square Error (RMSE) of 12% of the average yield when using weather data that had been predicted, and an RMSE of 11% of the average yield when using perfect weather data. Environmental data including weather and soil information and management practices were used as inputs to the CNN-RNN model developed by Khaki et al. (2020b) for corn and soybean yield prediction across the entire Corn Belt in the U.S. for the years 2016, 2017, and 2018. Their proposed CNN-RNN model outperformed other models tested including RF, deep fully connected neural networks, and LASSO Regression, achieving a notable improvement with an RMSE of 9% and 8% for corn and soybean average yields, respectively. They also employed a guided backpropagation technique to select features and enhance the model's interpretability. Similarly, Sun et al. (2019) adopted a comparable strategy, utilizing a CNN-LSTM model to predict county-level soybean yields in the U.S. using satellite imagery, climate data, and other socioeconomic factors. Their results show that the CNN-LSTM model can capture the spatiotemporal dynamics of soybean growth and outperform other models in terms of accuracy and computational efficiency. Oikonomidis et al. (2022) utilized a publicly available soybean dataset, incorporating weather and soil parameters to develop several hybrid deep learning-based models for crop yield prediction. Comparing their models with the XGBoost algorithm, the authors found that their hybrid CNN model outperformed the other models with an impressive RMSE of 0.266, Mean Squared Error (MSE) of 0.071, and Mean Absolute Error (MAE) of 0.199. However, none of these studies have addressed the issue of determining which crop genotype to plant based on the given weather conditions. The dataset, which was developed, prepared, and cleaned by Shook et al. (2021), provided us with unique genotype information on seeds, allowing us to investigate the potential of planting genotypes based on weather variables. Our proposed data-driven approach can be particularly valuable for selecting optimal genotypes when there are limited years of testing available. This is because the traditional approach of selecting the best genotypes based on a small number of years of field trials can be unreliable due to variations in weather and other environmental factors. By leveraging large datasets with genotype and weather information, it becomes possible to develop more accurate models that can predict the performance of different genotypes in various weather conditions. This can ultimately lead to the identification of genotypes that are both high-yielding and adaptable to different environments. Given that land for agriculture is limited, such data-driven approaches can help improve the productivity of crops per acre, as well as the quality and productivity of food crops through plant breeding.

This study aims to achieve three primary objectives. Firstly, it proposes two novel CNN architectures that incorporate a 1-D convolution operation and an LSTM layer. To achieve higher accuracy than other baseline models, the Generalized Ensemble Method (GEM) is utilized to determine the optimal weights of the proposed CNN-based models. Then, the Generalized Ensemble Method is utilized to select optimal genotypes for each location and weather condition. This is achieved by predicting the yield for all possible genotypes in each specific location and environmental scenario. This approach is evaluated using test dataset containing unseen genotype-location combinations, providing a simulation of scenarios where new genotypes are introduced. Secondly, the study assesses the impact of location, genotype, and weather variables on prediction outcomes, investigating critical time periods for weather variables in yield predictions throughout the growing season of 30 weeks. Lastly, the study investigates the impact of soil variables on Soybean yield prediction by incorporating state-level soil variables. Through these objectives, this study demonstrates the value of using data-driven approaches in plant breeding and crop productivity research.

The structure of this paper is as follows. Section 2 introduces the dataset used in this study. In Section 3, we propose a methodology for crop yield prediction and optimal genotype selection using two CNN-based architectures with a 1-D convolution operation and LSTM layer, as well as the GEM to find optimal model weights. This section also includes implementation details of the models used in this research, along with the design of experiments. Section 4 presents the experimental results, followed by an analysis of the findings in Section 5. Finally, in Section 6, we conclude the paper by discussing the contributions of this work and highlighting potential avenues for future research.

## 2 Data

### 2.1 Main dataset: MLCAS2021 Crop Yield Prediction Challenge

In this paper, the data analyzed was taken from the MLCAS2021 Crop Yield Prediction Challenge (MLCAS, 2021). The goal of the 2021 MLCAS Crop Yield Prediction Challenge was to predict soybean yield for the test data consisting of 10,337 performance records, using the training dataset containing 93,028 observations from all years and locations. Since the competition did not provide the ground truth response variables for the test data, our analysis in this paper relied solely on the training dataset, which comprises 93,028 samples from 159 locations across 28 states in the U.S. and Canadian provinces, over 13 years (2003–2015). The data included information on 5,838 unique genotypes and daily weather data for a 214-day growing season. This data was prepared and cleaned by Shook et al. (2021). The unique characteristic of this dataset is that it enables us to capture the biological interactions complexity, and temporal correlations of weather variables, as it provides both daily weather variables during the growing season for different locations and genotype data. The dataset included a set of variables for each performance record, which are as follows:

• Weather: Every performance record in the dataset included a multivariate time-series data for 214 days, which represent the crop growing season between April 1^st^ and October 31^st^. Each day in the record contained seven weather variables, including average direct normal irradiance (ADNI, Wm^-2^), average precipitation (AP, inches), average relative humidity (ARH, Percentage), maximum direct normal irradiance (MDNI, Wm^-2^), maximum surface temperature (MaxSur, °C), minimum surface temperature (MinSur, °C), and average surface temperature (AvgSur, °C). Records with the same location and yield year share the same set of weather variables.

• Maturity group: The dataset included 10 maturity groups corresponding to different regions.

• Genotype IDs: The dataset contained 5,838 distinct genotypes, which were further clustered into 20 groups using the K-means clustering technique as described in Shook et al. (2021). The resulting hard clustering approach allowed us to obtain a unique cluster ID for each of the 5,839 genotypes in the dataset.

• State: The state information was provided for each performance record, indicating the specific state that the record corresponds to. The data covers 28 U.S. states and Canadian provinces in total.

• Location ID: For each performance record, the dataset included the corresponding location ID, indicating the unique identifier for the location associated with the record. The data was collected from a total of 159 locations.

• Year: The performance record dataset contained information on the year when the yield was recorded, ranging from 2003 to 2015.

• Yield: The yield performance dataset included observed average soybean yields, measured in bushels per acre, from 159 locations across 28 U.S. states and Canadian provinces between 2003 and 2015. The data showed a mean yield of 50.66 bushels per acre and a median yield of 50.60 bushels per acre. Yield values ranged from a minimum of 0.4 to a maximum of 112.40 bushels per acre, with a standard deviation of 15.95. The 25th percentile was 39.8 bushels per acre, while the 75th percentile reached 61.40 bushels per acre.

**Figure 1:**
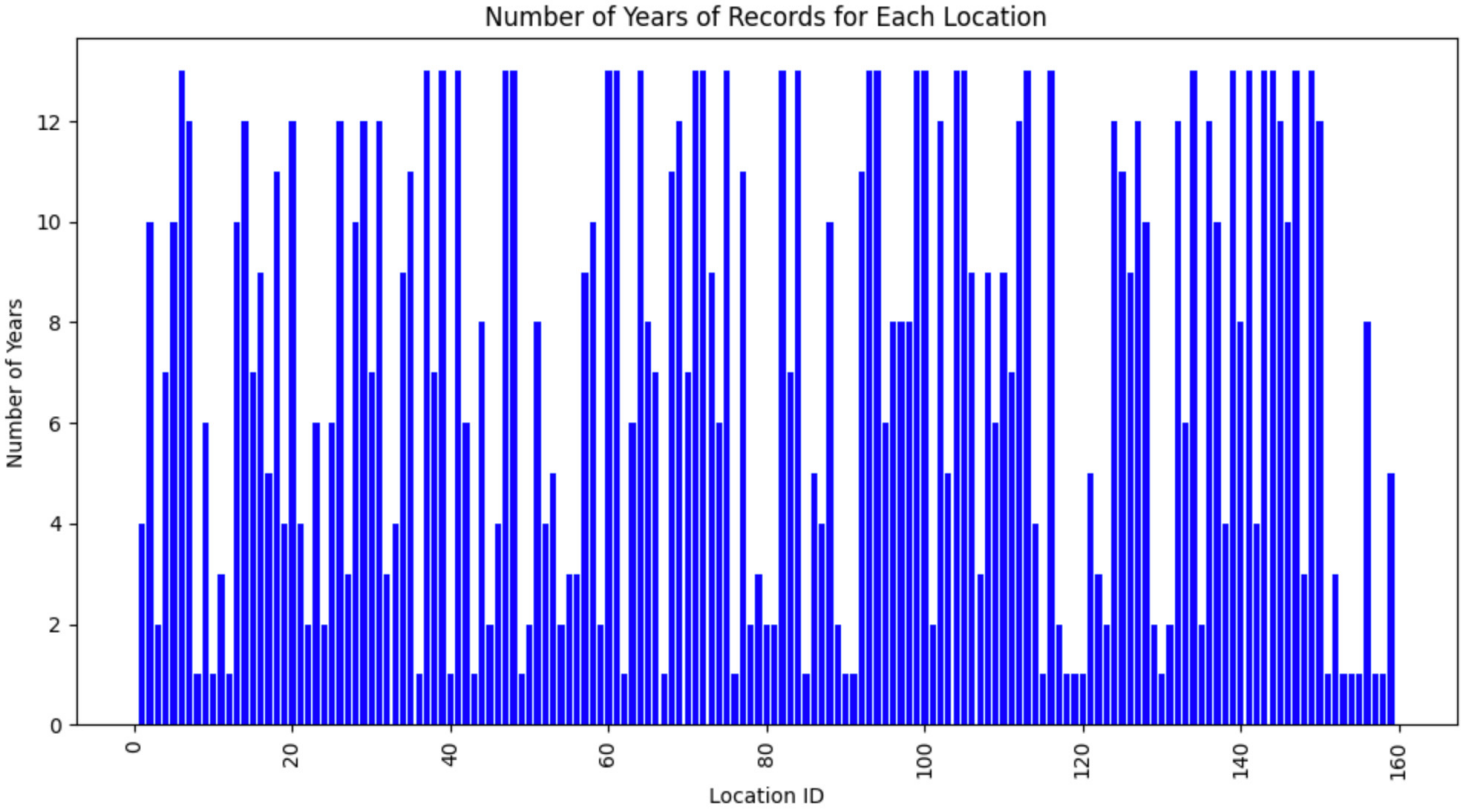
Temporal distribution of data availability for each location. The x-axis denotes the mapped location IDs (1–159), while the y-axis indicates the number of years with available weather records.

The dataset consisted of weather data gathered from 159 distinct locations across multiple years. Each location was identified by a unique location ID, which had been mapped to a continuous range from 1 to 159 for clarity. Figure 1 presents the temporal distribution of data availability for each location. The x-axis denotes the mapped location IDs (1–159), while the y-axis indicates the number of years with available weather records. As depicted in Figure 1, weather data for the 159 unique locations are not available for all years from 2003 to 2015. Some locations have data for only 1 year, others for 2 years, and so on.

**Figure 2:**
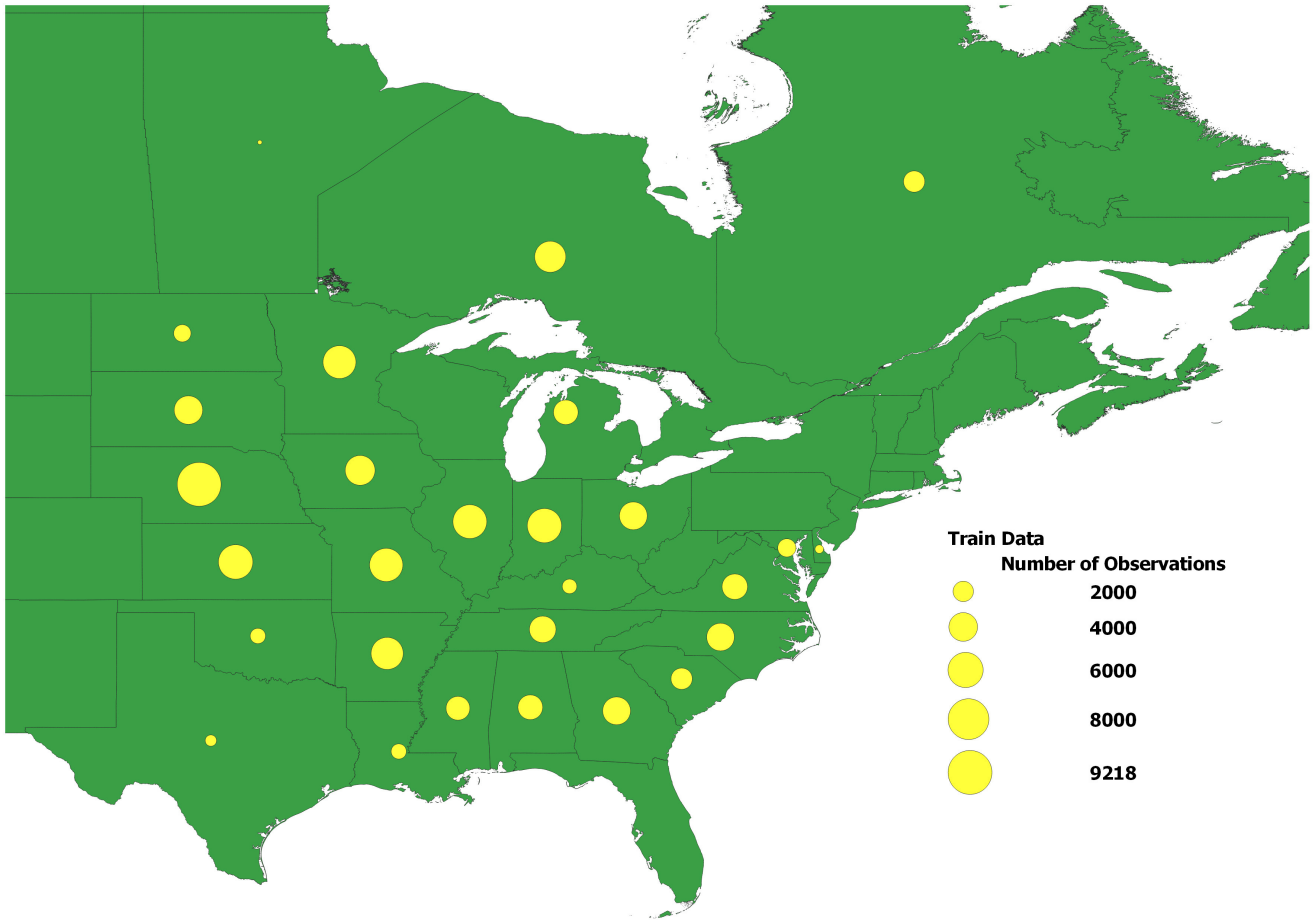
The distribution of performance records across 28 U.S. states and Canadian provinces in the dataset used in the study. The size of each yellow dot corresponds to the size of the dataset for the corresponding state\province.

Figure 2 displays the distribution of performance records across 28 U.S. states and Canadian provinces in this dataset. The size of each yellow dot corresponds to the size of the dataset for the corresponding state or province.

### 2.2 State-level soil data integration

To enhance our dataset with soil information, we utilized preprocessed and cleaned soil data available from an open-source repository on Github. The soil data originates from SoilGrids250m and comprises 11 variables measured at six different depths (0–5, 5–15, 15–30, 30–60, 60–100, and 100–200 cm) with a resolution of 250 m^2^ (Poggio et al., 2021; Turek et al., 2023). The corresponding acronyms and properties of these soil variables are listed in Table 1.

**Table 1:** Acronyms and corresponding soil properties.

**Acronym**	**Property**
bdod	Bulk density
cec	Cation exchange capacity at pH7
cfvo	Coarse fragments
clay	Clay
nitrogen	Total Nitrogen
ocd	Organic carbon density
ocs	Organic carbon stock
phh2o	pH in H2O
sand	Sand
silt	Silt
soc	Soil organic carbon

The soil data provided in the open-source repository includes average values of each soil variable across all counties within each state. This dataset encompasses soil information from both agricultural and non-agricultural areas, as it is not specifically limited to agricultural zones. To understand how soil data affects our analysis, we added 66 soil variables to each record in our dataset. We merged the soil data with our existing dataset using the State column. This ensures that all locations within the same state share the same soil information.

## 3 Method

### 3.1 Data preprocessing

The pre-processing tasks were conducted to ensure the data was in a useful and efficient format for fitting machine learning models. One of the main tasks involved one-hot encoding the categorical variables, which included year, location IDs, and genotype IDs. For the genotype data we tried both genotype clusters and the unique genotypes. The results demonstrated a considerable improvement when the genotype IDs were included with other variables. In one-hot encoding, each unique value of each categorical variable is represented as a new binary feature in a new column. This means that for every observation, a value of 1 is assigned to the feature that corresponds to its original category, while all other features are set to 0. This technique results in a new binary feature being created for each possible category, allowing for more accurate modeling and prediction. One-hot encoding facilitates accurate modeling because it prevents the model from making assumptions about any ordinal relationship between categories, which can occur if categorical variables are encoded with integers. By transforming categories into binary features, one-hot encoding ensures that categories are treated as independent, unrelated entities. This is especially useful for non-linear models, as it helps avoid misleading the model into inferring non-existent relationships between categories (Seger, 2018).

The weather data covers a total of 214 days, spanning the growing season from April 1^st^ through October 31^st^. To reduce the complexity of the daily weather data and make it more suitable for analysis, we aggregated the feature values by taking the average and downsampling the data to a 4-day level. We opted for 4-day intervals instead of the more commonly used weekly (7-day) intervals to better capture short-term variability in weather patterns that can significantly impact crop yield. Aggregating the data over 4 days provides a finer level of detail than weekly intervals, while still ensuring that the variation over each period is manageable and reliable. As a result of this downsampling and feature aggregation, we were able to reduce the number of model parameters significantly, with a dimension reduction ratio of 214:53. Reducing the daily weather data to a weekly level through downsampling has been commonly utilized in yield prediction studies to address the issue of excessive granularity in the data. This practice has been validated in prior research studies (Khaki and Wang, 2019; Shook et al., 2021; Srivastava et al., 2022).

Given the diverse range of values and varying scales of weather variables, it is important to avoid bias that may arise from a single feature. To address this, we applied the *z*-score normalization technique (Equation 1) to standardize all weather variable values. This technique rescales all weather variables to conform to a standard normal distribution, preventing any unintended bias on the results. In addition to mitigating bias, standardizing the weather variable values also improves the numerical robustness of the models and accelerates the training speed.


(1) $$\begin{array}{l} W_{i,j}=\frac{w_{i,j}-{\bar{w}}_{j}}{\sigma_{j}} \end{array}$$

where *W*_*i, j*_ is the standardized value of the *i*th observation of the *j*th weather variable (*j* ranges from 1 to *K*, where *K* represents the total number of weather variables, which in this case is 371 (7 variables * 53 time periods)), *w*_*i, j*_ is the original value of the *i*th observation of the *j*th weather variable, ${\bar{w}}_{j}$ is the mean of the *j*th weather variable, and σ_*j*_ is the standard deviation of the *j*th weather variable. The formula rescales each variable to have a mean of 0 and a standard deviation of 1.

To properly evaluate the proposed DL models and other ML models' performance on new and unseen genotype-location combinations, we carefully split the dataset into training, validation, and test sets. The dataset consists of 93,028 observations with 5,838 unique genotype IDs. Each observation includes information on genotype ID, year, location, and crop yield. To ensure the test set contains entirely new genotype-location combinations, we created a unique identifier for each combination of genotype and location. This identifier was crucial in making sure the model is trained on a diverse set of data while being evaluated on combinations it has never seen before. Initially, we split the unique genotype-location combinations into a training set (60%) and a temporary set (40%). The temporary set was further split into validation (20%) and test (20%) sets. This approach ensured that the validation and test sets were of equal size and helped in maintaining the balance of the dataset. After the initial split, we verified if all genotypes were present in the training set. This was necessary because having all genotypes in the training set is crucial for the model's ability to generalize. If any genotype was missing from the training set, we moved the first occurrence of that genotype from the validation or test set to the training set. This adjustment ensured that the model had exposure to all genotypes during training. Boolean masks were created for each split (train, validation, and test) based on the unique genotype-location combinations. These masks were then used to filter the original dataset into the respective training, validation, and test sets. This method allowed for a clear and precise division of the dataset, maintaining the integrity of the split process. We confirmed that there were no overlapping genotype-location combinations between the training, validation, and test sets. This was critical in ensuring that the test set contained entirely new combinations, providing a robust evaluation of the model's performance on unseen data. By removing any overlap, we aimed to mimic real-world scenarios where new genotypes are introduced.

The data preprocessing resulted in 6,381 column features (6,010 features after one-hot encoding year, location IDs, and genotype IDs, and 371 (53 × 7) features after downsampling the weather data to a 4-day level).

Table 2 provides detailed summary statistics for the training, testing, and validation datasets.

**Table 2:** Summary statistics of soybean yield for the training, testing, and validation datasets.

**Summary statistics**	**Train**	**Validation**	**Test**
Total number of locations	159	159	159
Year range	2003–2015	2003–2015	2003–2015
Mean yield	50.69	50.50	50.73
Standard deviation of yield	15.97	15.81	16.02
25th percentile of yield	39.8	39.7	39.9
Median yield	50.70	50.60	50.60
75th percentile of yield	61.40	61.10	61.50
Minimum yield	0.40	2.10	1.00
Maximum yield	112.40	106.70	110.60
Number of weather components	7	7	7
Number of genotype IDs	5,838	5,069	5,081
Number of observations	56,078	18,354	18,596

The unit of yield is bushels per acre.

### 3.2 Crop yield prediction model development

**Figure 3:**
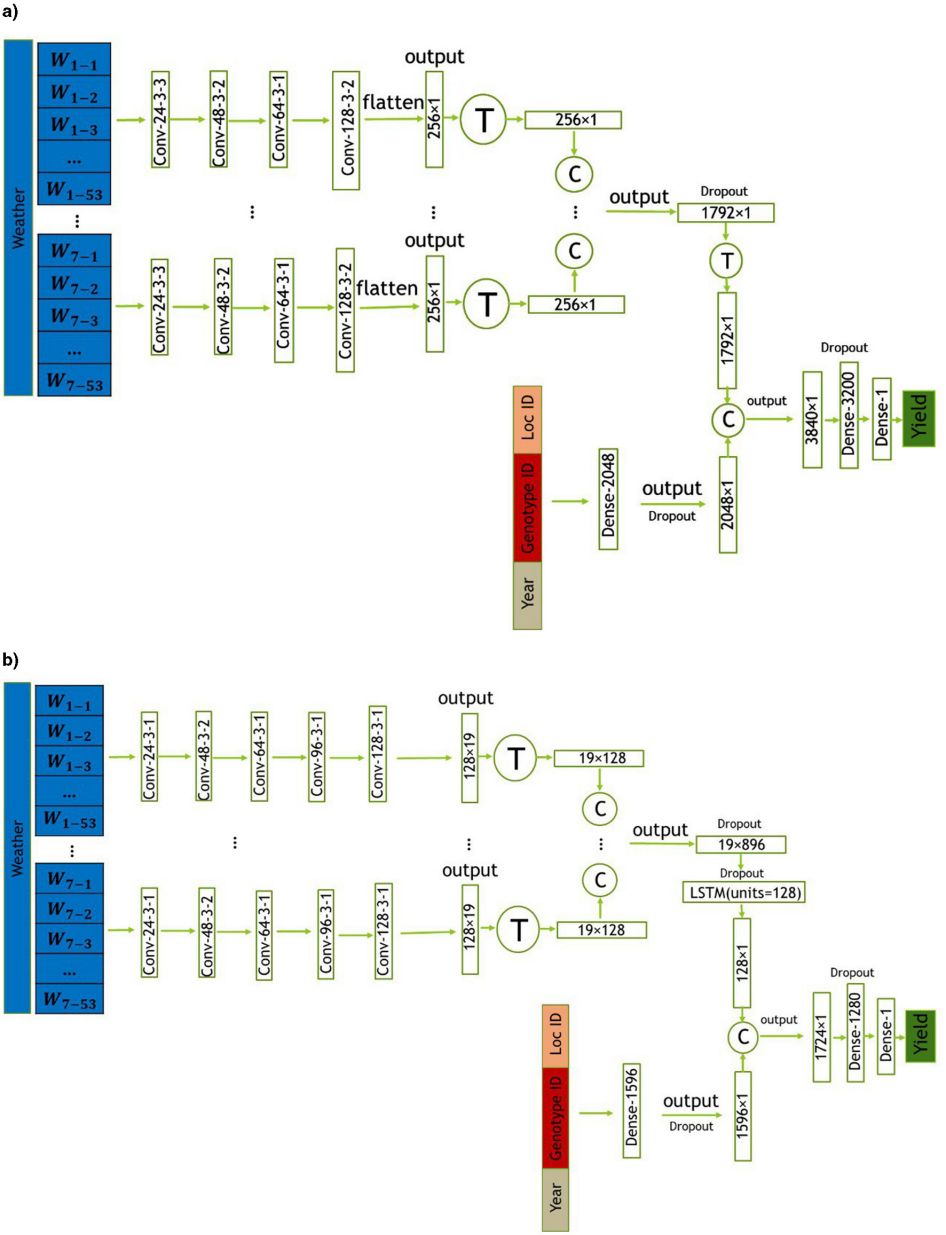
The CNN architectures proposed in this study includes convolutional, and fully connected layers denoted by Conv, and Dense, respectively. The parameters of the convolutional layers are presented in the form of “convolution type—number of filters—kernel size—stride size”. For all layers, “valid” padding was employed. Matrix concatenations are indicated by ©, while the symbol Ⓣ is used to indicate matrix transpose. Rectified Linear Unit (ReLU) was chosen as the activation function for all networks, with the exception of the fully connected layer in the input _other data, where a Leaky ReLU activation function was applied. **(a)** Proposed CNN model. **(b)** Proposed CNN-LSTM model.

**Figure 4:**
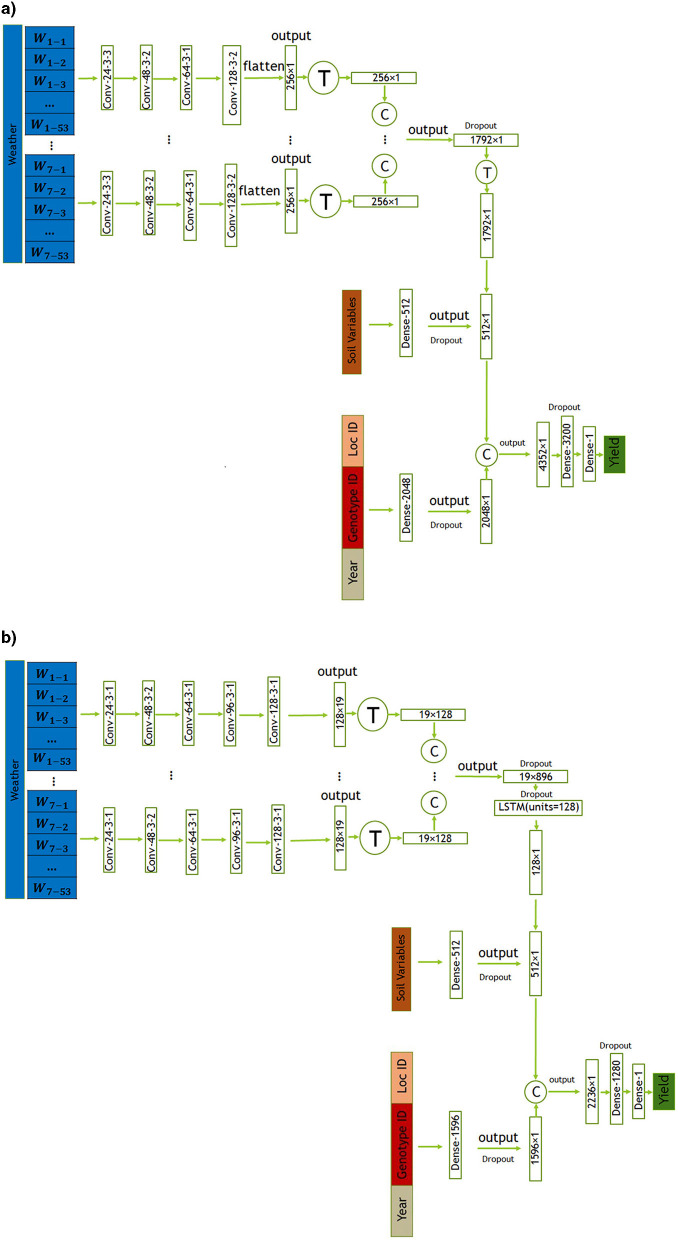
The CNN architectures proposed in this study includes convolutional, and fully connected layers denoted by Conv, and Dense respectively. The parameters of the convolutional layers are presented in the form of “convolution type—number of filters—kernel size—stride size”. For all layers, “valid” padding was employed. Matrix concatenations are indicated by ©, while the symbol Ⓣ is used to indicate matrix transpose. Rectified Linear Unit (ReLU) was chosen as the activation function for all networks, with the exception of the fully connected layers in the input _other data and soil data, where a Leaky ReLU activation function was applied. **(a)** Proposed CNN model incorporating soil data. **(b)** Proposed CNN-LSTM model incorporating soil data.

In this section, we introduce two proposed models, CNN and CNN-LSTM, for predicting crop yield using location, genotype, year, and weather data. These models are designed to handle the temporal features of weather data, which play a crucial role in crop yield prediction. CNN is a combination of CNNs and fully-connected (FC) neural networks, while CNN-LSTM is a combination of CNNs, LSTM networks, and FC neural networks. The architectures of the CNN and CNN-LSTM models are illustrated in Figures 3a, b, respectively. To evaluate the impact of integrating state-level soil data alongside the primary dataset, we incorporated this additional data into the architectures of both models. The modified versions of the CNN and CNN-LSTM models are illustrated in Figures 4a, b, respectively. All models were trained and evaluated using the same dataset for a consistent comparison.

The CNN model is simpler and faster to train due to its straightforward architecture, making it computationally efficient. Conversely, the CNN-LSTM model incorporates an LSTM layer after the CNN component to capture long-term temporal dependencies in the weather data.

To improve the accuracy of our yield predictions, we propose using the GEM method that combines the predictions of both models. This approach allows us to leverage the strengths of each model and obtain better RMSE values than either model alone. In the following subsections, we describe the architecture and training processes for the CNN and CNN-LSTM models, both with the primary dataset and the enhanced dataset that includes state-level soil variables. Additionally, we detail the implementation of the GEM method, used for yield prediction, to provide a comprehensive overview of our approach. We then outline our approach to feature importance analysis using RMSE change, followed by a description of the evaluation metrics applied in this study.

#### 3.2.1 Proposed CNN model

The first proposed model architecture combines CNNs and FC neural networks. The weather variables measured throughout the growing season are taken as input in the convolutional neural network part of the model, which captures their temporal dependencies, and linear and nonlinear effects through 1−dimensional convolution operations. The CNN part of the model takes in the seven weather variables separately and concatenates their corresponding output for capturing their high−level features. The data for genotype, location, and year (input _others) are fed into a fully−connected neural network with one layer. The high−level features from the CNN are then combined with the output of the fully−connected neural network for input _others data. The combined features are then processed through two additional FC layers before yielding the final prediction of the soybean yield. Moreover, to prevent overfitting, three dropout layers with dropout ratios of 0.5, 0.7, and 0.2 are respectively added to the fully connected layer after the CNN layer, at the end of the fully connected layer for input _others data, and at the final layer of the model. The proposed modeling architecture is designed to capture the complex interactions between weather data, genotype IDs, year, and location IDs for an accurate yield prediction and is illustrated in Figure 3a.

#### 3.2.2 Proposed CNN-LSTM model

The second proposed model is based on the architecture of the first one, but includes an LSTM layer at the end of the CNN part for the weather variables. Additionally, there are slight modifications in the CNN components and different numbers of neurons in the dense layers. Specifically, the output of the CNN part is passed to an LSTM layer consisting of 128 units. The resulting output is then combined with the output of the fully connected layer for the input _others data. This model architecture is designed to further capture the temporal dependencies and nonlinear effects of the weather variables, in addition to the high-level features extracted by the CNN part. Similar to the architecture described above, dropout layers are utilized to prevent overfitting. Four dropout layers with dropout ratios of 0.5, 0.5, 0.7, and 0.2 are respectively inserted after the CNN layer, at the LSTM layer, at the end of the fully connected layer for input _other data, and at the final layer of the model. The complete modeling architecture is illustrated in Figure 3b.

To ensure sufficient capacity for capturing intricate patterns in the combined inputs of genotype, location, and year, the dense layer in the CNN model has 2,048 neurons. This larger number of neurons compensates for the absence of sequential modeling through an LSTM layer, thereby enhancing the model's representational power. On the other hand, the CNN-LSTM model's dense layer has 1,596 neurons for the input_others data. By reducing the number of neurons in this layer, the overall complexity of the model is managed, which helps prevent overfitting and optimizes performance. Additionally, in the CNN model, the combined features are processed through a dense layer with 3,200 neurons, ensuring sufficient capacity to capture intricate patterns and interactions in the combined feature space. In contrast, the CNN-LSTM model processes the combined features through a dense layer with 1,280 neurons. The presence of the LSTM layer, which captures temporal dependencies in the weather data, reduces the need for a very large dense layer, resulting in a more balanced and efficient model architecture.

#### 3.2.3 Integrating state-level soil variables in CNN and CNN-LSTM models for improved crop yield prediction

As highlighted in Subsection 2.1, the dataset lacks information about soil variables for each location. The available data only included location IDs and states, with no provided latitude and longitude details. Consequently, the exact geographical coordinates for each location ID were unavailable, limiting our spatial information to state-level knowledge. In this section, we aim to investigate the impact of incorporating state-level soil data in addition to the variables provided by the MLCAS2021 Crop Yield Prediction Challenge.

The employed CNN and CNN-LSTM models, detailed in Subsections 3.2.1 and 3.2.2 respectively, remained consistent with the methodology outlined in this paper. The key modification involved integrating soil data via an additional dense layer. This layer, structured with 512 neurons, processes the input soil data using a Leaky ReLU activation function. To prevent overfitting and enhance the model's robustness, dropout regularization was applied. This regularization technique randomly dropped out approximately 50% of the neurons during training, preventing the model from relying too heavily on specific features and aiding in better generalization. The architectural configurations of both the CNN and CNN-LSTM models, incorporating soil data, are visually represented in Figures 4a, b, respectively.

#### 3.2.4 Generalized Ensemble Method

The Generalized Ensemble Method is an advanced technique for creating a regression ensemble that combines the strengths of multiple base estimators. The method was first proposed by Perrone and Cooper (1995) in the context of artificial neural networks. The main goal of GEM is to find the optimal weights of the base models that minimize the error metric, such as MSE or RMSE. To prepare the data for model training and evaluation, we partitioned the dataset into a training set containing 56,078 samples, a validation set including 18,354 samples, and a test set containing 18,596 samples. We selected the best performing model on the validation set and leveraged the following optimization approach to create an ensemble of models that further improved the prediction accuracy. The problem can be stated as a nonlinear convex optimization problem, where the objective is to minimize the sum of squared errors between the true values (*y*_*i*_) and the predicted values (ŷ_*ij*_) of all observations (*i*= 1…, *n*) by the *k* base models (*j* = 1,…, *k*). The validation set was used to optimize the ensemble weights.


(2) $$\underset{w_{j}}{\min}\frac{1}{n}\sum_{i=1}^{n}{\left(y_{i}-\sum_{j=1}^{k}w_{j}{\hat{y}}_{ij}\right)}^{2}$$

The problem is subject to two constraints: the weights of all base models should be non-negative (*w*_*j*_≥0) and sum up to one ($\overset{k}{\underset{j=1}{\sum }}\left(w_{j}\right)=1$). Here, *w*_*j*_ represents the weight assigned to base model *j*.

#### 3.2.5 Feature importance analysis using RMSE change

In this study, we conducted a feature importance analysis to identify the key predictors that significantly influence our model's predictions. The analysis was based on the RMSE change, which measured the impact of feature permutations on prediction performance. This method allowed us to assess the impact of variable shuffling on the model's performance.

• **Baseline RMSE calculation**: We initially computed the baseline RMSE (*r0*) using the proposed GEM model predictions (*yhat*) and the ground truth values (test set containing 18,596 samples).

• **Permutation and RMSE change**: We systematically shuffled the columns within various groups of variables and recalculated the RMSE for each permutation. These groups encompassed variables related to weather conditions, such as ADNI, AP, ARH, MDNI, MaxSur, MinSur, and AvgSur. Additionally, we considered other critical variables, including year, location, and genotype ID. Among these, the categorical variables, such as year, location, and genotype IDs, underwent one-hot encoding, resulting in multiple variables representing these categories. Similarly, each weather-related variable comprises 53 distinct variables, each signifying the aggregation of daily feature values through the process of averaging and downscaling the data to a 4-day granularity.

• **Interpreting RMSE change**: A higher RMSE change after shuffling indicated that the original group of variables had a more substantial impact on the model's predictions. In other words, when these variables were shuffled, the model's performance degraded significantly because they were contributing substantially to the model's accuracy. Conversely, a lower RMSE change after shuffling suggested that the original group of variables had a lesser influence on the model's predictions. Shuffling these variables did not significantly impact the model's performance, indicating that they might not be as critical for prediction accuracy.

#### 3.2.6 Model evaluation

In this study, we evaluated the performance of our prediction models using two widely used metrics: MAE (Equation 3) and RMSE (Equation 4). Both of these metrics provide a measure of the distance between the predicted and actual values of the target variable. Specifically, MAE represents the average absolute difference between the predicted and actual values, while RMSE represents the square root of the average of the squared differences between the predicted and actual values. By using both of these metrics, we were able to assess the accuracy of our models and compare their performance against each other. We also reported the results of correlation coefficient (*r*) (Equation 5) as an additional metric to evaluate the linear relationship between the predicted and actual values.


(3) $$\text{MAE}=\frac{1}{n}\sum_{i=1}^{n}|y_{i}-{\hat{y}}_{i}|$$

(4) $$\text{RMSE}=\sqrt{\frac{1}{n}\sum_{i=1}^{n}{\left(y_{i}-\hat{y_{i}}\right)}^{2}}$$

(5) $$r=\frac{\sum_{i=1}^{n}\left(y_{i}-\bar{y}\right)(\hat{y_{i}}-\bar{\hat{y}})}{\sqrt{\sum_{i=1}^{n}{\left(y_{i}-\bar{y}\right)}^{2}\sum_{i=1}^{n}{\left(\hat{y_{i}}-\bar{\hat{y}}\right)}^{2}}}$$

Where *n* is the total number of data points, *y*_*i*_ is the true value of the *i*-th data point, ŷ_*i*_ is the predicted value of the *i*-th data point, and ȳ and $\bar{ŷ}$ represent their respective means.

### 3.3 Optimal genotype by environment selection

The aim of optimal genotype by environment selection is to identify the best genotypes for cultivation in each specific location and weather scenario. To achieve this, we utilized the entire dataset to identify the top 10 genotypes with the highest yields for each location-environment combination.

To assess our approach, we split the data into training, validation, and test sets, ensuring that the test dataset consisted of entirely new genotype-location combinations that are not present in the training or validation sets. This approach ensures that the model is trained on a diverse set of data while being evaluated on unseen genotype-location combinations, thereby mimicking real-world scenarios where new genotypes are introduced. Based on the results, the GEM model demonstrated superior performance on unseen genotype-location combinations. Consequently, we proceeded to retrain the proposed CNN, CNN-LSTM, and GEM models using the entire dataset.

As described in Subsections 3.2.1 and 3.2.2, the inputs of the base models include weather data, genotype, location, and year. We excluded the maturity group variable because different types of maturity groups were utilized in each location. Following this, the model was used to predict yields for all 5,838 genotypes for each record with its specific weather and location information.

Next, we chose the top 10 genotypes with the highest yields. We then proceeded to compute the average yield for these elite genotypes across each location-environment combination. As illustrated in Figure 1, the weather data available for each location varied significantly. Specifically, not all locations had weather data spanning the full thirteen years. Consequently, the amount of weather data differed from one location to another. For example, for location ID 167, we selected the top 10 genotypes with the highest yields based on weather variables from 2008 to 2010. In contrast, for location ID 163, we selected the top 10 genotypes with the highest yields based on weather variables from 2004 to 2012.

## 4 Results

In this section, we begin by evaluating the proposed GEM model's performance in predicting yields for new and unseen genotype-location combinations, using both the primary dataset and the dataset integrated with state-level soil variables. We then present the results of the feature importance analysis, providing insights into the key factors driving yield predictions. Finally, we use the model to predict yields across all 5,838 genotypes, utilizing specific weather and location data for each record, and discuss the optimal genotype selections that achieved the highest yields for each specific location and environmental condition.

### 4.1 Prediction results for soybean yield using deep learning and machine learning models on unseen genotype-location combinations

In order to make a comprehensive comparison, we incorporated three additional commonly used prediction models: Random Forest (RF; Breiman, 2001), Extreme Gradient Boosting (XGBoost; Chen and Guestrin, 2016), and Least Absolute Shrinkage and Selection Operator (LASSO) Regression (Tibshirani, 1996). Further details on the implementation of these models are outlined in Appendix A. We maintained the same partitioned dataset, which was used to train the ensemble models, consisting of a training set with 56,078 samples, a validation set with 18,354 samples, and a test set with 18,596 samples, for both hyperparameter tuning and model evaluation. Multiple models were trained using various hyperparameter values and their performance was evaluated on the validation set. The hyperparameter values that resulted in the best performance on the validation set were selected, and the corresponding model was evaluated on the test set to estimate its generalization performance. The range of hyperparameter values that we tested was selected based on our domain knowledge. Table 3 shows the tested hyperparameters along with the best estimates obtained for the baseline models. The architecture and hyperparameters of the CNN and CNN-LSTM models are described in Figures 3a, b, respectively. We trained the proposed models using the Adam optimizer with a scheduled learning rate of 0.0004, which decayed exponentially with a rate of 0.96 every 2,500 steps. The models were trained for 100,000 iterations with a batch size of 48. ReLU was chosen as the activation function for all networks, with the exception of the fully connected layers in the input _other data and soil data, where a Leaky ReLU activation function was applied.

**Table 3:** Hyperparameters of the baseline machine learning models employed to predict soybean yield.

**Model**	**Parameters**	**Best parameter**
RF	Number of estimators	650
	Max. feature numbers	Sqrt
	Max. depth	55
	Min. samples split	5
	Min. samples leaf	1
	Bootstrap	FALSE
XGBoost	Max. depth	13
	Objective	[reg:squared error]
	regularization alpha	0.0001
	Min. child weight	5
	Gamma	0.05
	Learning rate	0.09
	Booster	Gbtree
	Subsample	0.5
	Column sample by tree	0.9
LASSO Regression	alpha	0.0001

The performance of the proposed CNN, CNN-LSTM, and GEM models in predicting soybean yield is evaluated based on RMSE, MAE, and the correlation coefficient (r), with results presented in Table 4. The performance of the baseline machine learning models is detailed in Supplementary Table 1, located in Appendix A.

**Table 4:** Comparison of test and validation results of RMSE, MAE, and r for the proposed CNN, CNN-LSTM, and GEM models in predicting soybean yield.

	**CNN**	**CNN-LSTM**	**GEM**
Train RMSE	2.683	2.535	-
Validation RMSE	7.633	7.779	7.613
Test RMSE	7.688	7.855	7.674
Train MAE	1.772	1.969	-
Validation MAE	5.787	5.939	5.779
Test MAE	5.848	6.018	5.844
Train r	0.987	0.986	-
Validation r	0.876	0.871	0.877
Test r	0.878	0.872	0.878

The units of both RMSE and MAE are bushels per acre, matching the units of the predicted and actual yield values.

The optimal weights for the ensemble were determined to be 0.744 for CNN and 0.256 for CNN-LSTM, indicating that the CNN model contributed more to the final prediction. Integrating the base models through the GEM ensemble approach demonstrated much better performance compared to the CNN-LSTM base model and slightly better performance compared to the CNN model in terms of all evaluation metrics on the validation and test sets. The ensemble approach might have benefited from combining more base models. The relatively small differences between validation and test set performance for the GEM model suggest that it is not overfitting significantly. The strong correlation coefficients (r) obtained for all models indicate a robust relationship between predicted and actual yields.

The performance of the proposed GEM model was compared with benchmark machine learning models, including XGBoost, Random Forest, and LASSO Regression. Based on the results shown in Supplementary Table 1, the GEM model outperformed all other tested models. It is because, the GEM model combines the strengths of multiple models, including the highly nonlinear structure of the CNN model and the ability to capture the temporal dependencies of weather data using the LSTM model. This results in a more robust and accurate model that outperforms the other tested models. Specifically, the GEM model achieved a reduction in RMSE of 5.264%, 3.896%, and 24.798%, and a reduction in MAE of 3.302%, 2.583%, and 27.567% compared to RF, XGBoost, and LASSO Regression, respectively, on the test data. Among the baseline ML models, XGBoost and RF demonstrated comparable performance, both outperforming linear models by effectively capturing the nonlinear relationships between variables. LASSO, on the other hand, is a linear regression model with an L1 penalty, which can result in some of the coefficients being forced to zero. While this can result in a simpler and more interpretable model, it may not be able to capture the complex relationships present in the weather data, and genotype by environment interactions. Despite extensive hyperparameter tuning using train-validation-test splits to select the best hyperparameters based on validation RMSE and MAE, some models, such as CNN, CNN-LSTM, and Random Forest, still exhibit overfitting, as indicated by the noticeable differences between training and validation/test performance metrics.

#### 4.1.1 Impact of state-level soil characteristics on soybean yield prediction

We maintained consistency in our experimental setup by employing identical training, test, and validation datasets, as well as the same set of hyperparameters for traditional ML models, as outlined in Subsections 3.1 and 4.1. For our proposed CNN and CNN-LSTM models, we maintained consistency by using the same learning rate, number of iterations, batch size, and activation functions, as detailed in Subsections 4.1. Additionally, after incorporating soil variables into the existing data, we conducted a comparison of the models' performances. The results of this comparison are presented in Table 5, shedding light on the impact of integrating soil variables into the predictive models. Given the lack of precise latitude and longitude data for each location, we resorted to using state-level soil data. Therefore, the inclusion of soil variables as additional input features did not yield substantial improvements in model performance, which aligns with our expectations. Noteworthy is the marginal impact observed in the RMSE for the Test data. Specifically, the RMSE decreased by only 2.15%, and 0.32% for the CNN-LSTM, and GEM models, respectively. In contrast, the CNN model did not show any improvement; instead, its performance slightly worsened, with an increase of 0.27% in RMSE. The comparison of the ML models performance is presented in Supplementary Table 1, located in Appendix A. This table demonstrates that the RMSE for the XGBoost model decreased by just 0.21%, while the RMSE for the RF and LASSO Regression models showed negligible changes, with reductions of 0.03% and 0.02%, respectively. These minimal improvements highlight the inherent challenges of effectively incorporating soil data. As a result, despite our efforts, the additional granularity provided by the soil data did not significantly enhance the performance of the models.

**Table 5:** Comparison of test and validation results of RMSE, MAE, and r for the proposed CNN, CNN-LSTM, and GEM models with and without soil variables in predicting soybean yield.

**Model**	**No soil variables**			**All 66 soil variables are included**		
	**RMSE**	**MAE**	**r**	**RMSE**	**MAE**	**r**
**Train**						
CNN	2.683	1.772	0.987	4.861	2.467	0.943
CNN-LSTM	2.535	1.969	0.986	1.742	1.302	0.994
GEM	-	-	-	-	-	-
**Validation**						
CNN	7.633	5.787	0.876	7.638	5.779	0.876
CNN-LSTM	7.779	5.939	0.871	7.667	5.799	0.875
GEM	7.613	5.779	0.877	7.602	5.746	0.878
**Test**						
CNN	7.688	5.848	0.878	7.709	5.847	0.877
CNN-LSTM	7.855	6.018	0.872	7.686	5.837	0.878
GEM	7.674	5.844	0.878	7.649	5.804	0.879

The units of both RMSE and MAE are bushels per acre, matching the units of the predicted and actual yield values.

### 4.2 Feature importance analysis using RMSE change

**Figure 5:**
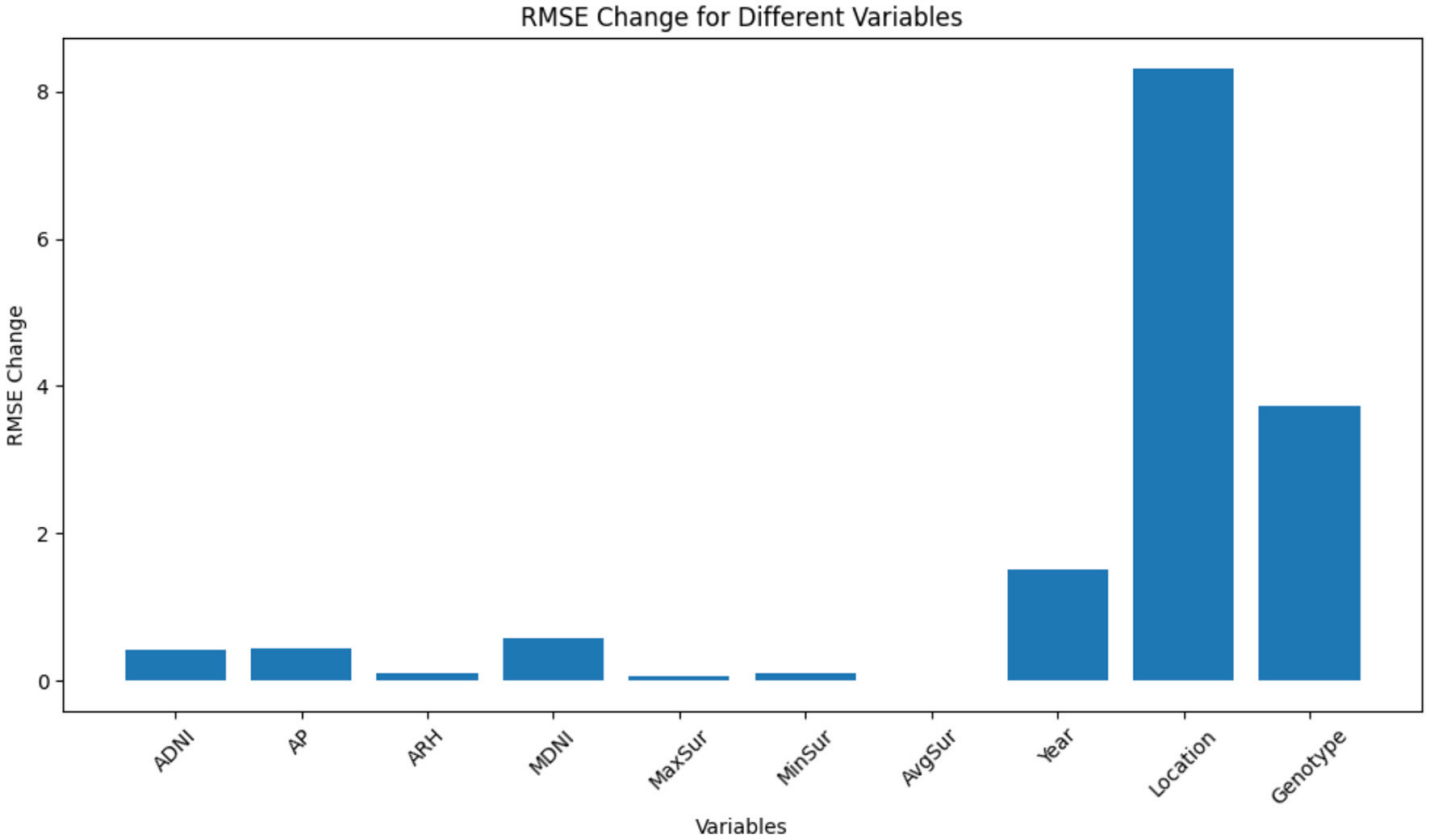
The RMSE changes for different groups of variables after shuffling. Each group represents a set of variables, and the RMSE change quantifies the impact of shuffling those variables on the model's predictions.

Figure 5 illustrates the RMSE changes for different groups of variables after shuffling. Each group represents a set of variables, and the RMSE change quantifies the impact of shuffling those variables on the model's predictions.

Among all the groups, the location variable exhibits the highest RMSE change, suggesting that it plays a pivotal role in the model's predictions. When the location variable is shuffled, there is a significant decline in model performance. This emphasizes the substantial influence of geographical location on prediction accuracy. Following location, the genotype variable shows the second-highest RMSE change. Its shuffling causes a notable decrease in model accuracy, underscoring its importance in achieving reliable predictions. Different genotypes or plant varieties evidently contribute significantly to the model's predictive power. The year variable ranks third in terms of RMSE change. Shuffling the year variable leads to a substantial drop in model performance. This implies that variations across different years significantly affect the model's ability to make accurate predictions, likely due to year-specific climate patterns or other time-dependent factors. Within the weather category, MDNI demonstrates the highest RMSE change, followed by AP, ADNI, MinSur, ARH, MaxSur, and AvgSur. While these weather-related variables do influence the model's predictions, their impact appears to be less pronounced compared to the location, genotype, and year variables. Shuffling these weather variables results in a relatively modest effect on model performance, suggesting that they may be less critical for prediction accuracy compared to the aforementioned groups.

### 4.3 Optimal genotype selection

The differences between the average predicted yield of optimal genotypes and the actual yield of existing genotypes for each year with specific weather variables for each location were calculated and are shown in Supplementary Figure 3. Each year and state contains a number of unique locations ranging from 1 to 10. The observed differences across all years suggest that the optimal genotypes can potentially lead to increased average soybean yields in all states, with improvements ranging from 5.1 to 42.5 bushels per acre.

These visualizations underscore the nuanced influence of weather conditions on the selection of optimal genotypes for achieving the highest yields. Moreover, they emphasize the critical role of genotype choice in varying weather conditions. For instance, consider Location ID 1, located in the state of Louisiana (LA). Depending on the weather variables corresponding to the year, the top 10 genotypes for the highest predicted yields varied, exemplifying the sensitivity of optimal genotype selection to different weather conditions. Significantly, the impact of weather variables on achieving the highest yield with optimal genotypes is magnified when considering diverse Location IDs, states, and provinces. The variability in weather variables for different years also contributes to the observed differences in yield outcomes. For example, in the province of Manitoba (MB), which had weather data for only one location in the years 2003, 2004, 2005, 2006, 2007, 2010, and 2011, the original data showed that 2004 had the lowest yields for the 14 genotypes present in the dataset. Correspondingly, the prediction results for 2004 also exhibited the lowest range compared to other years. This led to the highest differences between the average predicted yield of optimal genotypes and the actual yield of existing genotypes among all states and years. Notably, the absence of weather variables for certain years in specific location IDs adds another layer of complexity. Illustratively, for weather variables corresponding to the year 2004, the range of differences spans from 6.04 for the state of DE (Delaware) to 42.5 for the province of MB (Manitoba). In contrast, for weather variables corresponding to the year 2010, the range narrows to 11.05 for the state of IA (Iowa) and 23.73 for the province of MB (Manitoba). The highest differences between the average predicted yields of optimal genotypes and the actual yields of existing genotypes are inherently linked to the specific weather variables corresponding to different years. It is noteworthy that the years 2014 and 2015 exhibit a lower number of locations in our analysis. It is interesting that the difference generally increases with latitude other than Arkansas, adding a compelling layer of geographical insight to our findings.

## 5 Discussion

**Figure 6:**
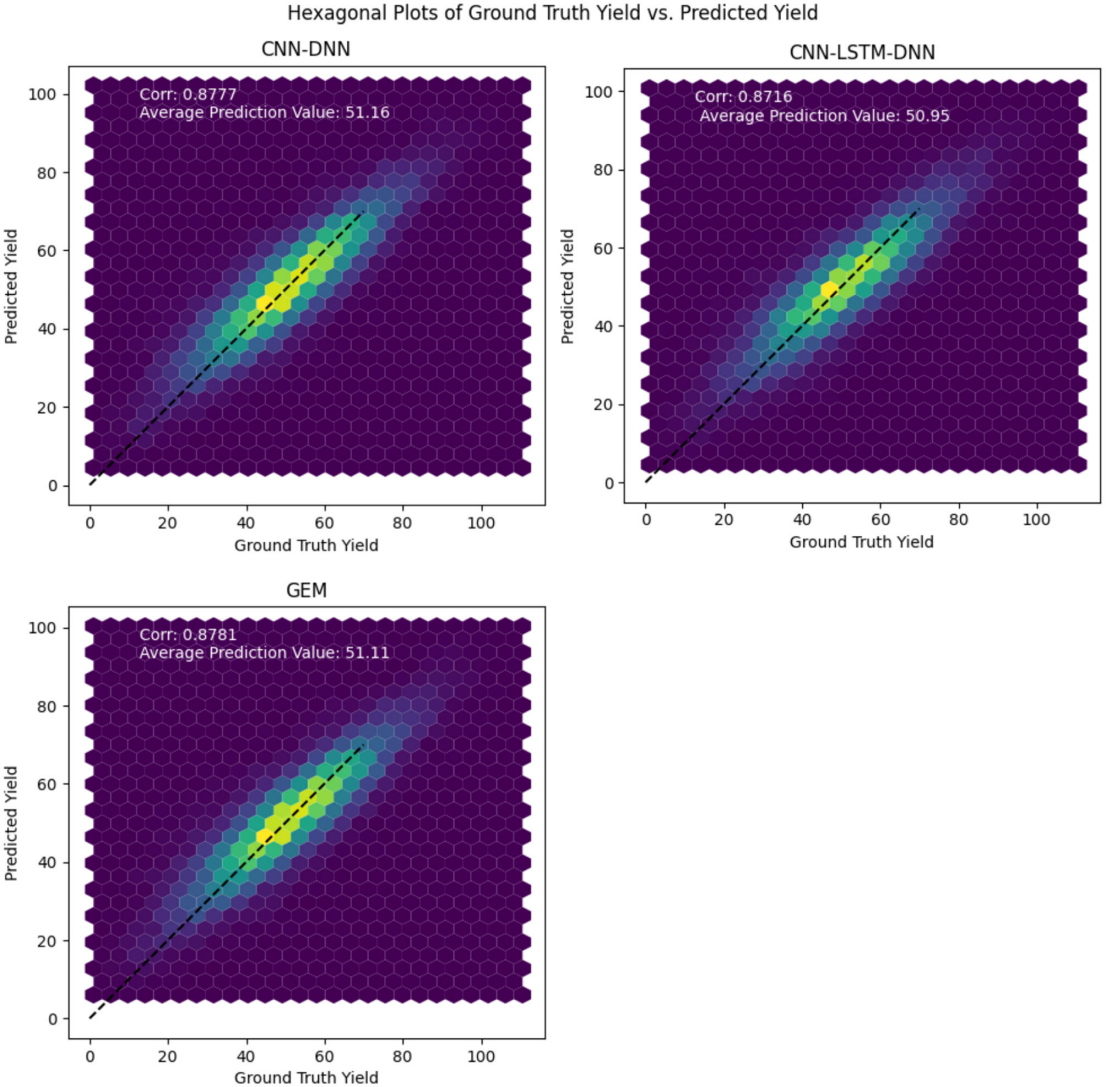
Hexagonal plots of the predicted soybean yield vs. ground truth yield values for the proposed CNN, CNN-LSTM, and GEM models on the test data.

The hexagonal plots shown in Figure 6 are a visualization tool used to compare the ground truth yield with the predicted yield values for the proposed models. The plots show the density of points where the two yields overlap, with the color of the hexagons representing the density of points. The 1:1 line represents the ideal situation where the predicted yield is exactly equal to the ground truth yield.

By looking at the hexagonal plots and the position of the points relative to the 1:1 line, we can observe how well each model is performing. If the points are concentrated near the 1:1 line, it indicates that the model is performing well, with high accuracy and precision. On the other hand, if the points are scattered or far from the 1:1 line, it indicates that the model is not performing well and is making large errors in its predictions.

The hexagonal plots in Figure 6 indicate strong overall predictive performance for all models, as demonstrated by a dense clustering of points around the diagonal line. Notably, the GEM model exhibits a slightly tighter point distribution, suggesting potentially greater reliability across the yield range and improved precision in error reduction. While all models show some scatter, particularly at lower yield levels, indicating potential challenges in predicting low yields, their performance is more consistent in the mid to high yield ranges.

In our analysis, we observed that certain time periods within the weather variables exhibited the highest RMSE change after shuffling. Specifically, for two key weather variables, Maximum Direct Normal Irradiance (MDNI) and Average Precipitation (AP), we identified the time periods that demonstrated the most significant impact on model performance.

For MDNI, we found that time period 25, which corresponds to approximately week 15^th^, exhibited the highest RMSE change following shuffling, as it is shown in Supplementary Figure 1. Similarly, for AP, time period 19 (approximately week 10^th^) showed the highest RMSE change, as illustrated in Supplementary Figure 2. These findings prompt us to explore the relationship between these time periods and the growth stages of soybeans in the United States.

In the context of soybean growth in the U.S., the growth stages are often categorized into Vegetative (V) and Reproductive (R) stages. Based on our analysis and considering typical soybean growth patterns in the USA (McWilliams et al., 1999; University of Kentucky Cooperative Extension, 2023), we can provide the following insights:

• Week 10 (Approximately): During this time, soybeans are in the early to mid-vegetative stages, typically ranging from V4 to V6. They are transitioning from early vegetative growth to the onset of reproductive growth (University of Kentucky Cooperative Extension, 2023).

• Week 12 (Approximately): At this stage (mid to late June), soybeans are typically in the V6 to V8 vegetative stage, indicating that they are approaching the reproductive stages (University of Kentucky Cooperative Extension, 2023).

• Week 15 (Approximately): This period, occurring in early to mid-July, corresponds to soybeans being in the V8 to V10 vegetative stage. This is a critical time when soybeans start transitioning to early reproductive stages, with some plants beginning to flower (R1 stage; University of Kentucky Cooperative Extension, 2023).

• Week 17 (Approximately): Around mid to late July, soybeans may have progressed to the R2 (Full Flower) to R3 (Beginning Pod) stages. This is a vital phase during which soybeans flower and initiate pod development (University of Kentucky Cooperative Extension, 2023).

The observed highest RMSE changes in time periods 25 (MDNI) and 19 (AP) suggest a noteworthy correlation with soybean growth stages. The marked sensitivity of soybean growth to solar radiation (MDNI) during the transition from vegetative to reproductive stages underscores the importance of adequate sunlight in meeting the energy needs of the plants as they begin to flower. Similarly, the high sensitivity to precipitation (AP) during the early vegetative stages highlights the critical role of sufficient water supply in supporting vegetative growth and preparing the plants for subsequent stages. These findings underscore the significance of weather variables during crucial growth phases of soybeans and their influence on accurate yield predictions.

## 6 Conclusion

In this study, we proposed two novel CNN architectures that incorporate a 1-D convolution operation and an LSTM layer. These models were developed to predict soybean yield using a combination of factors, including genotype ID, year, location, and weather data. Our study is based on an extensive dataset collected from 159 locations across 28 U.S. states and Canadian provinces over a span of 13 years. These architectures represent an advancement in the field of crop yield prediction, allowing us to leverage the power of deep learning to improve accuracy and efficiency in genotype selection. Moreover, we have employed the GEM method to determine the optimal weights of our proposed CNN-based models, which has led to superior performance in the MLCAS2021 Crop Yield Prediction Challenge and compared to baseline models.

Our work has gone beyond traditional crop yield prediction methods by addressing the challenge of genotype by environment interaction, which is a critical factor in selecting genotypes for increased crop yields, particularly in the face of global climate change. Conventionally, plant breeders rely on extensive field testing of hybrids to identify those with the highest yield potential, a process that is both time-consuming and resource-intensive. Our approach has introduced a data-driven paradigm for genotype selection, wherein we use environmental data and genotype information to predict crop yields. This approach enables us to identify the most efficient genotypes for each location and environmental condition by forecasting crop yields based on weather conditions and then selecting the optimal genotype with the highest yield. This novel strategy holds the potential to significantly enhance policy and agricultural decision-making, optimize production, and ensure food security.

To validate the effectiveness of our proposed GEM model for genotype by environment selection, we conducted a comprehensive evaluation alongside other DL and ML models. We specifically focused on new and unseen genotype-location combinations to mimic real-world scenarios where new genotypes are introduced. Our dataset, comprising 93,028 observations with 5,838 unique genotype IDs, was meticulously split into training, validation, and test sets to ensure robust assessment. Unique identifiers for each genotype-location pair were created to guarantee the test set contained entirely new combinations. This approach not only tested the model's predictive accuracy but also its generalizability to novel situations. The results demonstrated that the GEM model outperformed traditional methods. Specifically, it exhibited lower RMSE and MAE values ranging from 3.89% to 24.79% and 2.58% to 27.56%, respectively, compared to the baseline models when evaluated on test data. Additionally, the GEM model showcased higher correlation coefficients ranging from 1.14% to 8.66% in comparison to the baseline models on test data. These performance improvements suggest the effectiveness of the GEM model in soybean yield prediction, attributed to its ability to capture the nonlinear nature of weather data and model the temporal dependencies of weather variables, including genotype by environment interactions. This is achieved through the combination of two CNN-based models, which are adept at handling complex relationships in the data.

We retrained the CNN, CNN-LSTM, and GEM models using the complete dataset, predicting yields for 5,838 genotypes across each location with specific weather information. By selecting the top 10 genotypes with the highest predicted yields, we observed that optimal genotypes could potentially increase average soybean yields across all states, with improvements ranging from 5.1 to 42.5 bushels per acre. The analysis highlighted the significant influence of weather conditions on genotype selection and the model's ability to adapt to varying environmental conditions. Variability in weather data across locations and years, as well as the general increase in yield differences with latitude, underscored the model's robust performance and potential for enhancing agricultural decision-making.

Additionally, we conducted a feature importance analysis using RMSE change to identify significant predictors affecting the model's predictions. The location variable had the highest RMSE change, indicating its strong influence on predictions. Genotype, and year also played crucial roles. Among weather variables, MDNI had the most impact, followed by AP, ADNI, and others. While weather variables influenced predictions, categorical variables like location and genotype were more influential.

In addition to evaluating the importance of different variable groups, we explored the temporal aspects of weather data. We identified significant time periods within the MDNI and AP variables that showed the highest RMSE changes after shuffling. Notably, the highest RMSE changes were observed in time periods 25 (week 15) for MDNI and 19 (week 10) for AP. These findings underscore the critical roles of solar radiation and precipitation in plant development, emphasizing the importance of adequate sunlight and water supply during specific growth phases for accurate yield predictions.

Despite the constraints imposed by limited information and the absence of exact latitudes and longitudes in our dataset, we opted to explore the impact of soil variables on model performance. We accommodated this limitation by integrating state-level soil variables into the original dataset. Our findings suggest that the integration of soil variables, under the current data constraints, did not lead to a substantial enhancement in the predictive capabilities of the models. Given that climate change can also have an adverse effects on soil attributes (Das et al., 2016), it is advisable to consider datasets that provide precise soil variables for each specific location. This more granular data can significantly enhance our understanding of the intricate relationships among weather, soil, and crop outcomes. The incorporation of location-specific soil attributes into predictive models has the potential to elevate accuracy, particularly in regions where soil quality plays a pivotal role in agricultural outcomes.

## Data Availability

Publicly available datasets were analyzed in this study. This data can be found at: https://github.com/tryambakganguly/Yield-Prediction-Temporal-Attention.
