# Supplementary material for: A hybrid deep learning-based approach for optimal genotype by environment selection
Source: Front Artif Intell. 2024 Dec 11;7:1312115. doi: 10.3389/frai.2024.1312115 (PMC11670329; doi:10.3389/frai.2024.1312115)
Supplement: Supplementary file 2 [file Data_Sheet_2.docx]

Additional Results

•RF is an ensemble learning algorithm in machine learning. It works by combining multiple decision trees and using bagging to create a more accurate model. Bagging involves randomly sampling the dataset multiple times with replacement, creating different subsets of data for each tree to learn from. Each tree is trained on a different subset of the data, and their predictions are combined to make the final prediction. After experimenting with various numbers of trees in the RF model, we discovered that 650 trees produced the most accurate predictions. Furthermore, increasing the number of trees did not improve the accuracy but significantly increased the training time. We also examined different numbers of maximum tree depths and observed that a maximum depth of 55 generated the most precise predictions. Altering the maximum depth of the trees had a significant impact on the prediction accuracy, with an increase resulting in overfitting and a decrease leading to decreased prediction accuracy.

•XGBoost is a popular machine learning algorithm known for its speed and accuracy in solving regression and classification problems. It is based on the concept of boosting, where weak learners are combined to form a strong learner. XGBoost is an optimized version of gradient boosting, and it uses a tree-based model. It has several advantages, such as handling missing values, feature importance ranking, and regularization to prevent overfitting. To optimize the XGBoost model for predicting soybean yield, we explored different hyperparameters ranges for max depth and subsample. After training and validating multiple models with different combinations of hyperparameters, we found that a max depth of 13 and a subsample of 0.5 provided the best results in terms of RMSE and MAE.

•LASSO is a linear regression technique used to analyze data with a high number of features. It uses regularization to constrain the coefficient estimates towards zero, which results in simpler models and reduces the risk of overfitting. The LASSO Regression model adds a penalty term to the sum of the squared residuals, where the penalty is proportional to the absolute value of the coefficients. The optimization algorithm tries to minimize this penalty term along with the sum of the squared residuals. The alpha parameter in sklearn’s LASSO function controls the strength of the L1 penalty on 20 arXiv Template A PREPRINT the coefficients, which is the same as the L1 term in the LASSO Regression model. A higher alpha value will result in more coefficients being forced to zero, leading to a simpler and more interpretable model. In our study, we tried a range of alpha values, including [0.0001, 0.001, 0.01, 0.1, 1, 10, 100], and found that alpha=0.0001 provided the best result.
