# Supplementary figures and images for: A hybrid deep learning-based approach for optimal genotype by environment selection
Source: Front Artif Intell. 2024 Dec 11;7:1312115. doi: 10.3389/frai.2024.1312115 (PMC11670329; doi:10.3389/frai.2024.1312115)

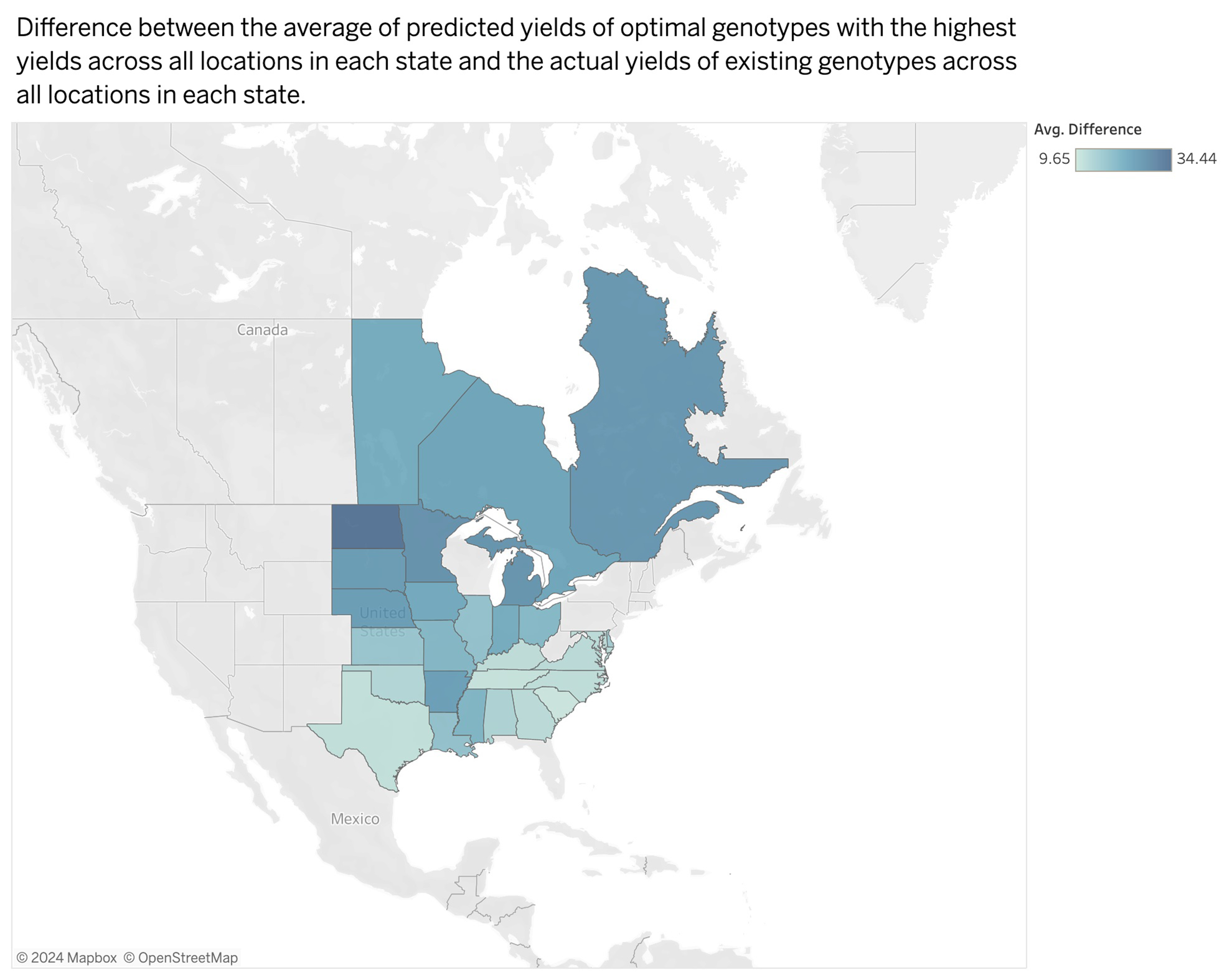

Supplement: Supplementary file 3 [file Image_1.jpeg]

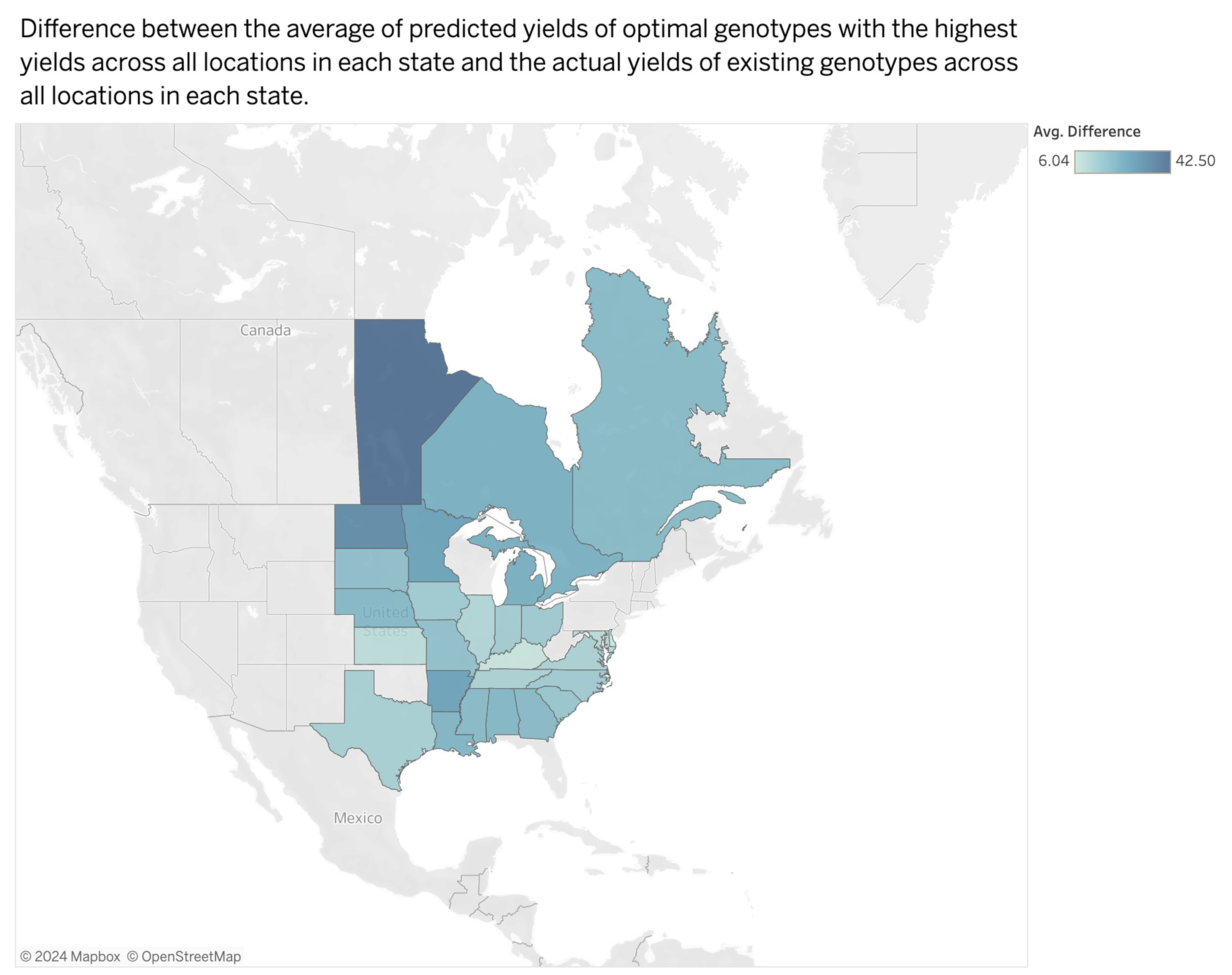

Supplement: Supplementary file 4 [file Image_2.jpeg]

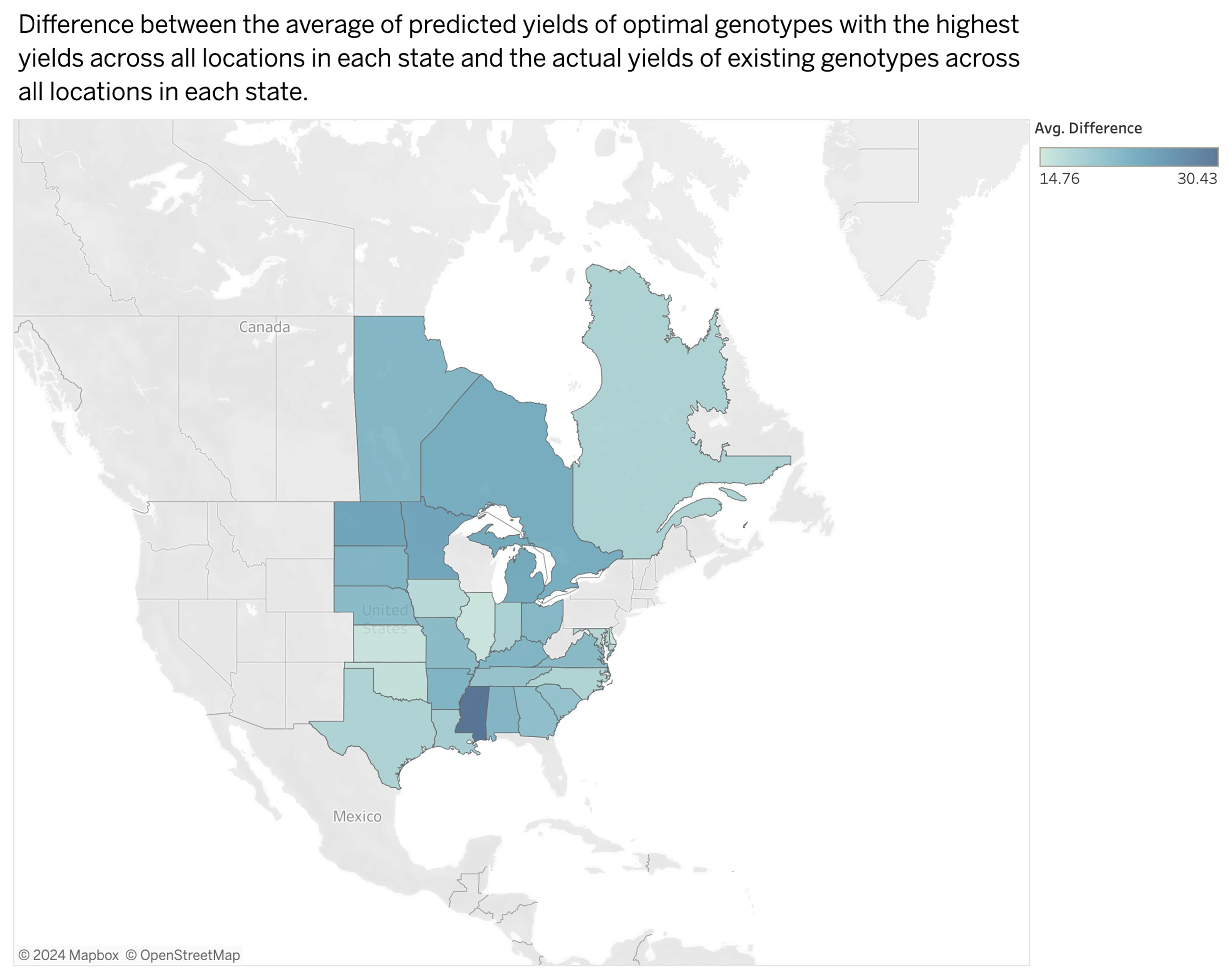

Supplement: Supplementary file 5 [file Image_3.jpeg]

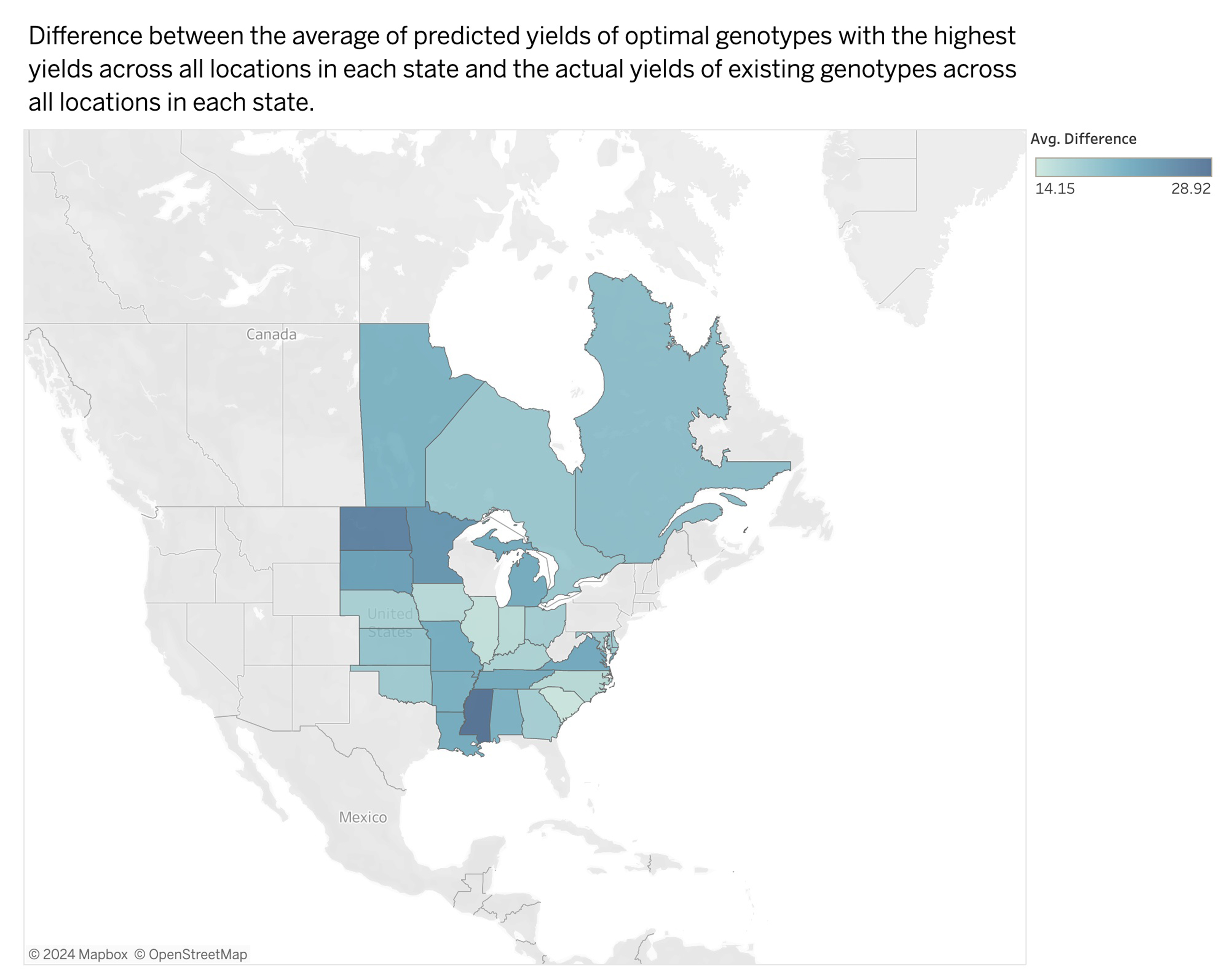

Supplement: Supplementary file 6 [file Image_4.jpeg]

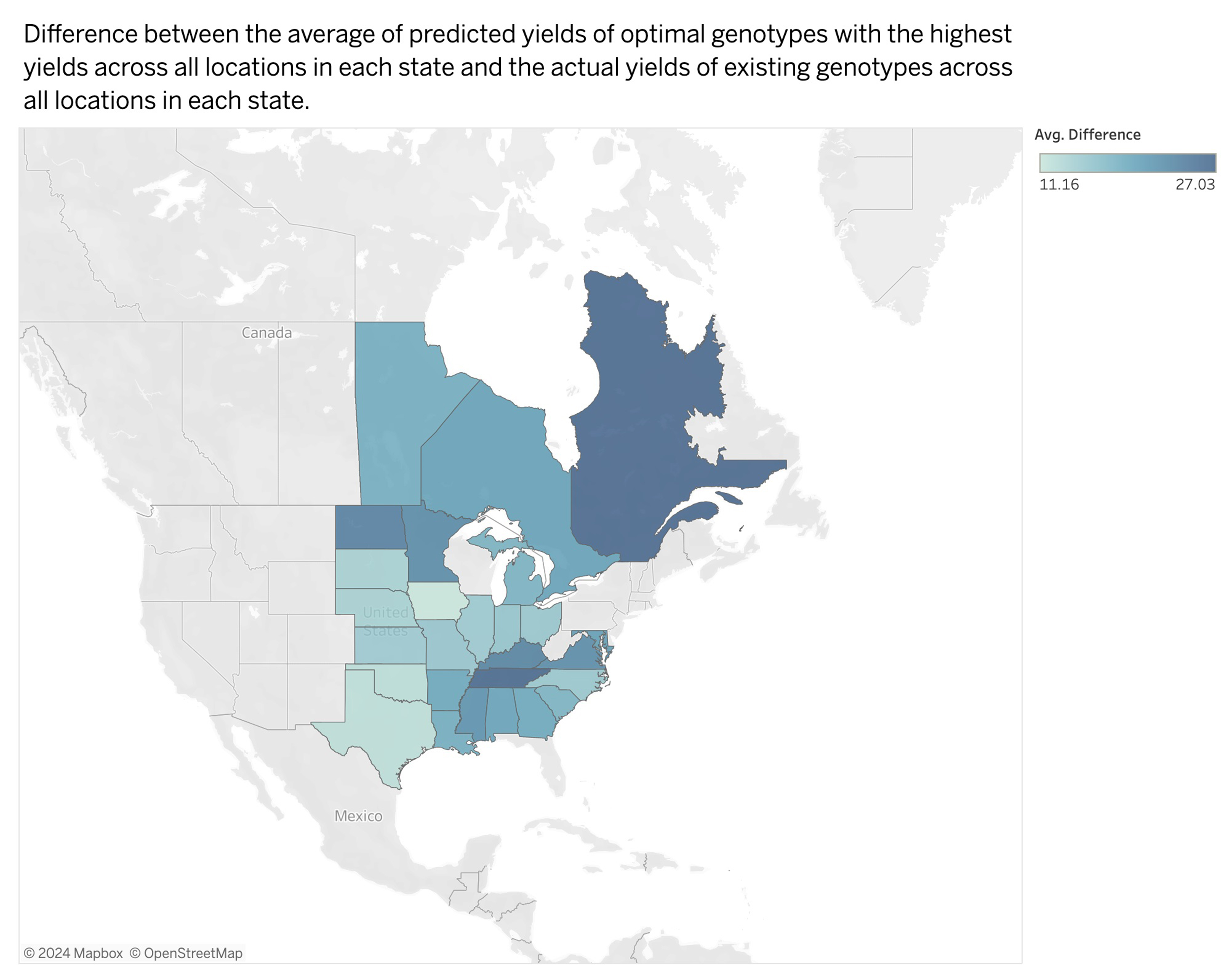

Supplement: Supplementary file 7 [file Image_5.jpeg]

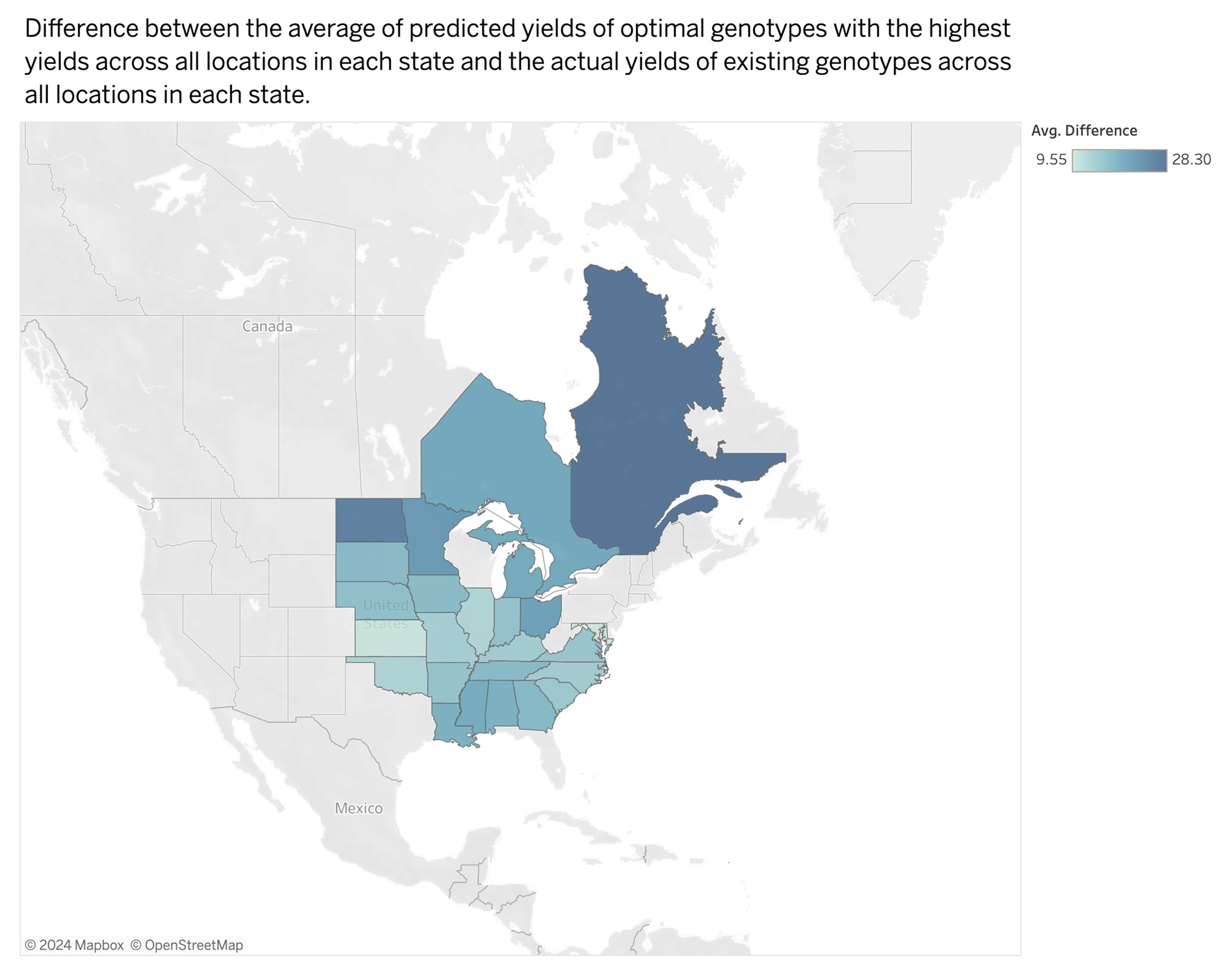

Supplement: Supplementary file 8 [file Image_6.jpeg]

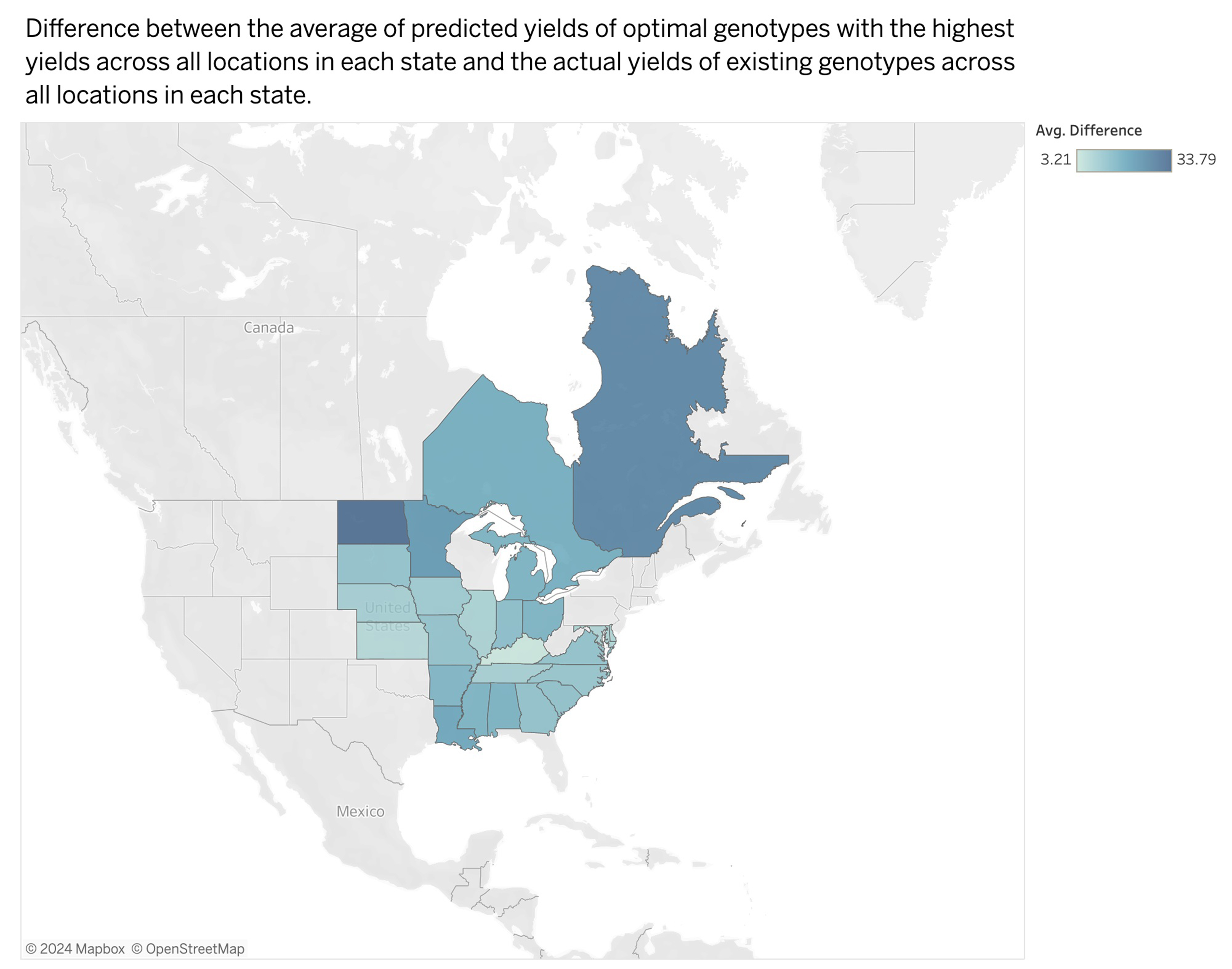

Supplement: Supplementary file 9 [file Image_7.jpeg]

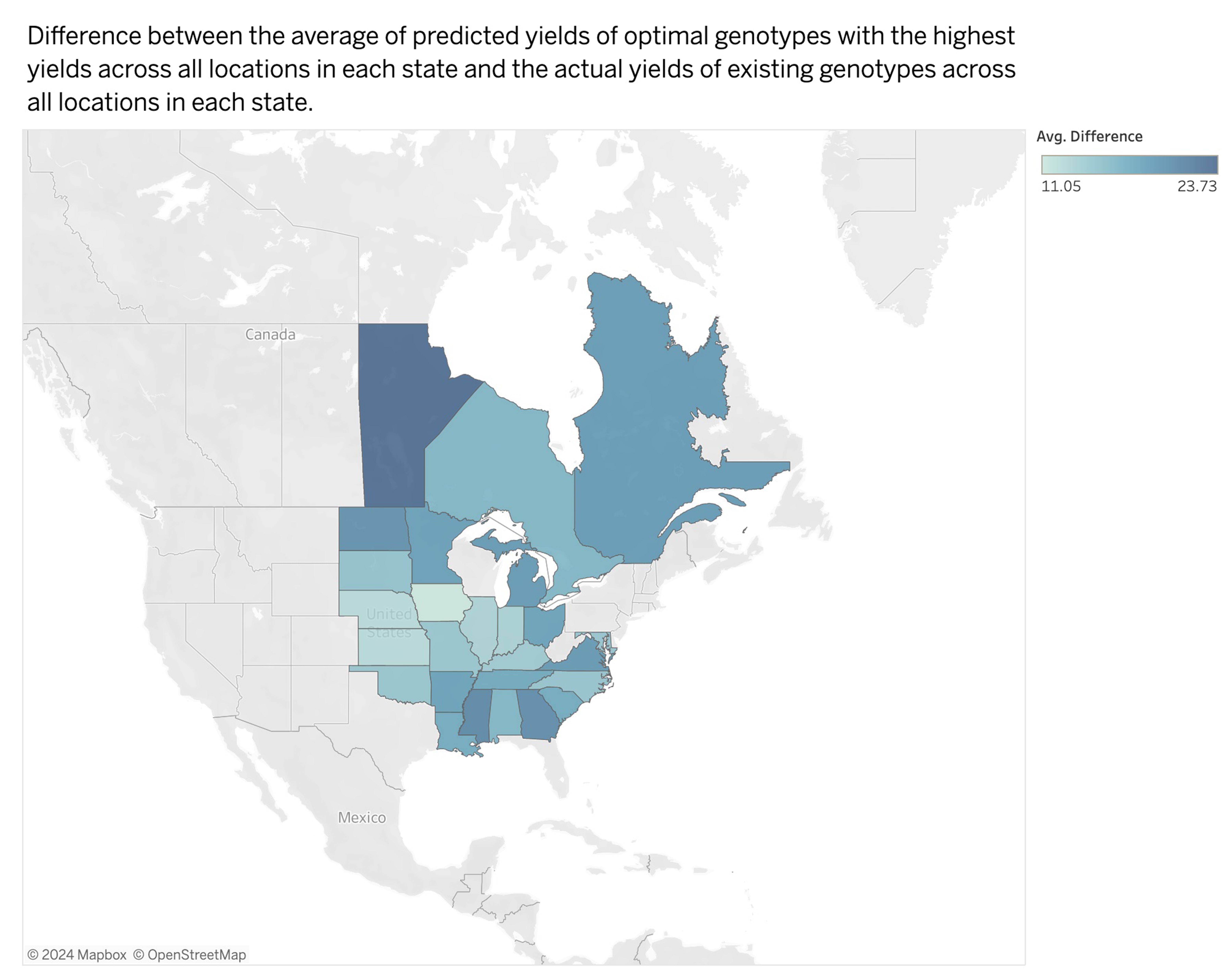

Supplement: Supplementary file 10 [file Image_8.jpeg]

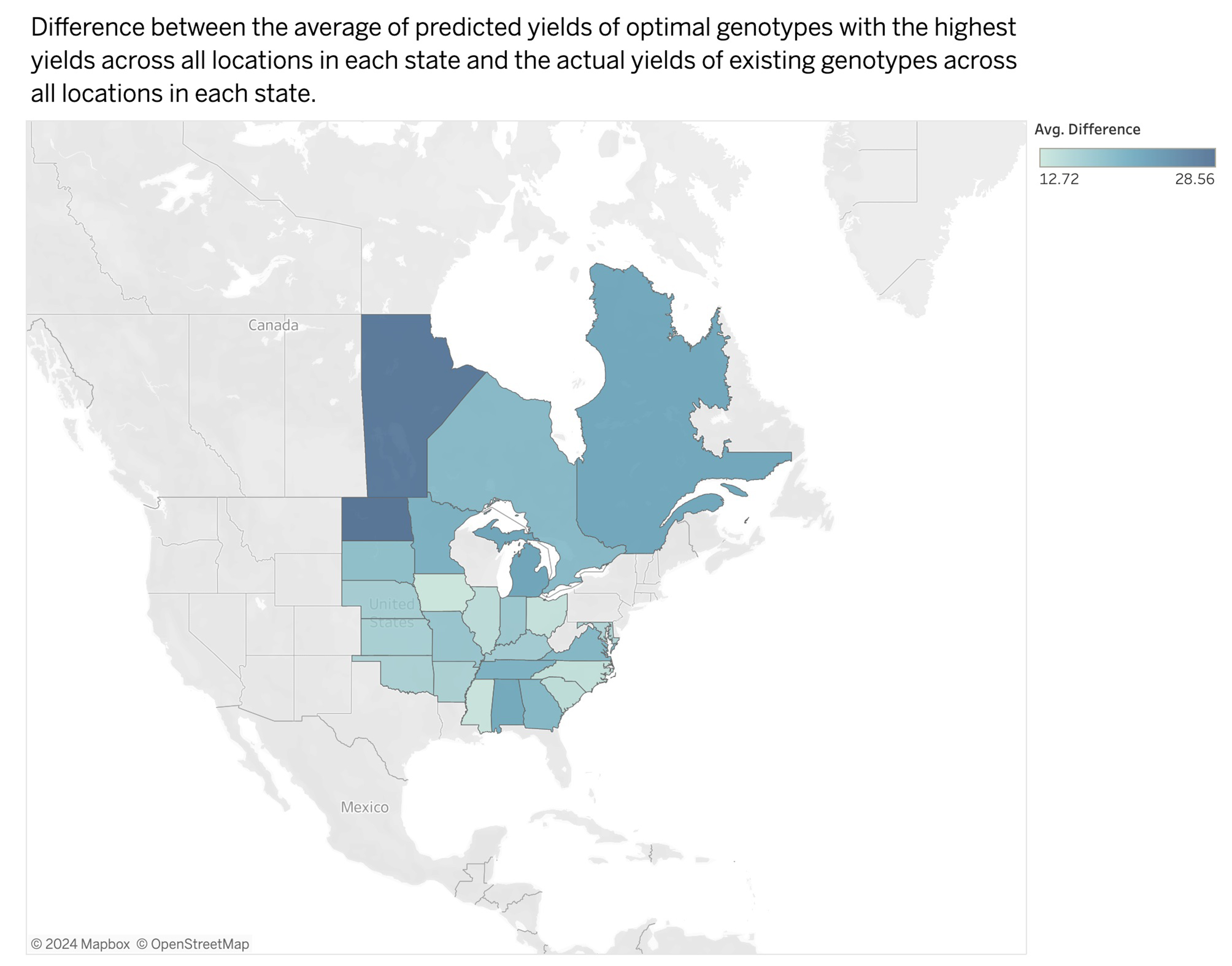

Supplement: Supplementary file 11 [file Image_9.jpeg]

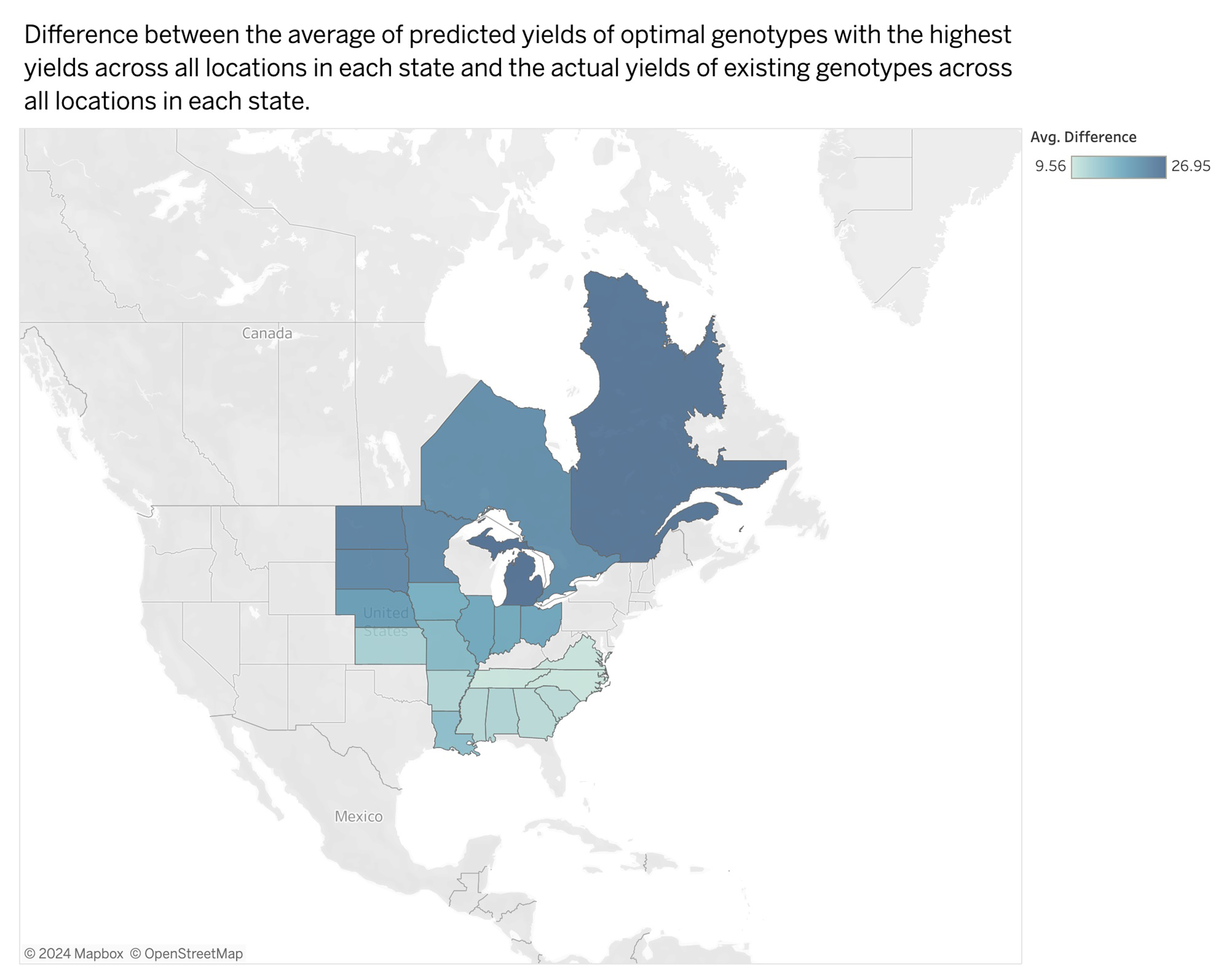

Supplement: Supplementary file 12 [file Image_10.jpeg]

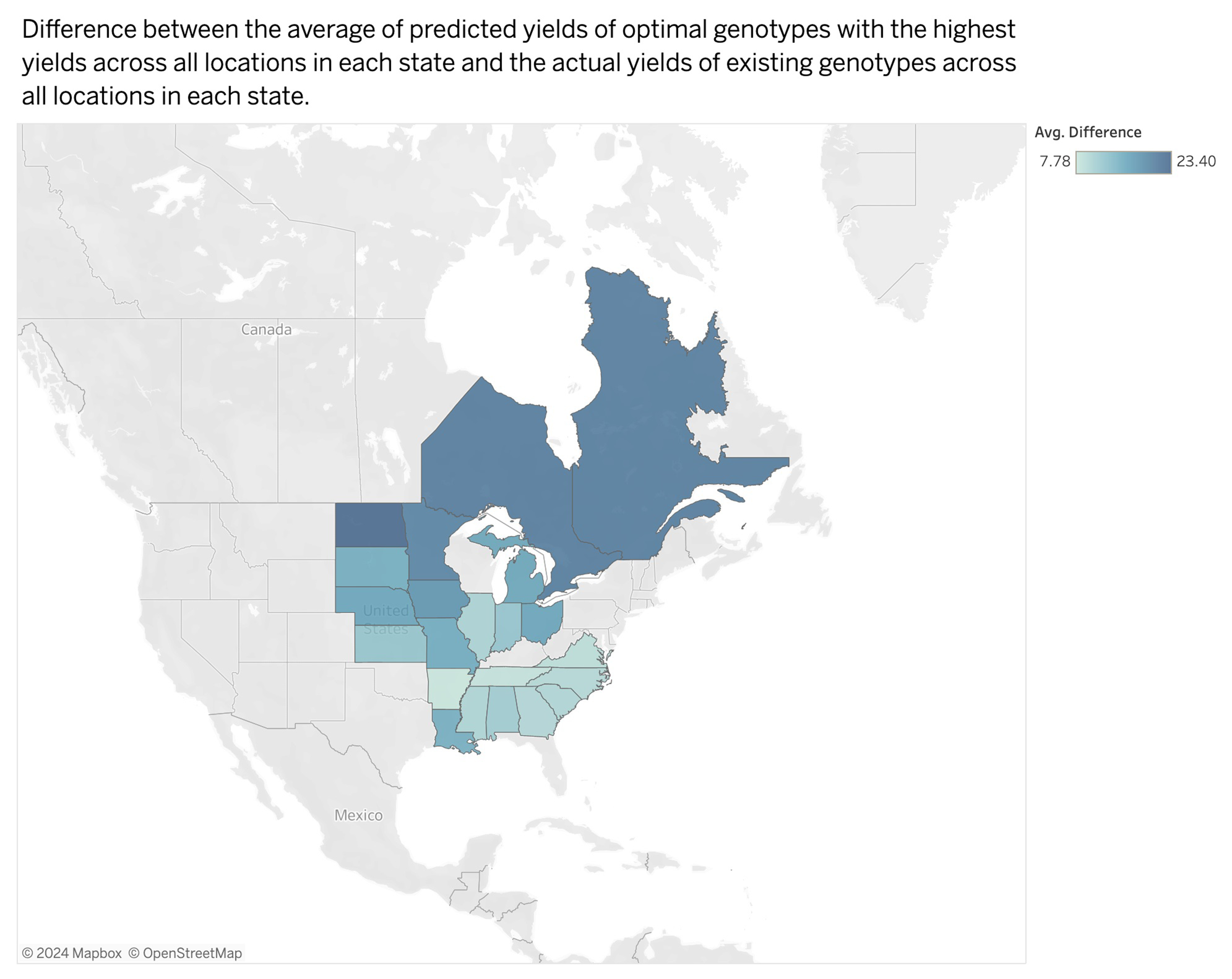

Supplement: Supplementary file 13 [file Image_11.jpeg]

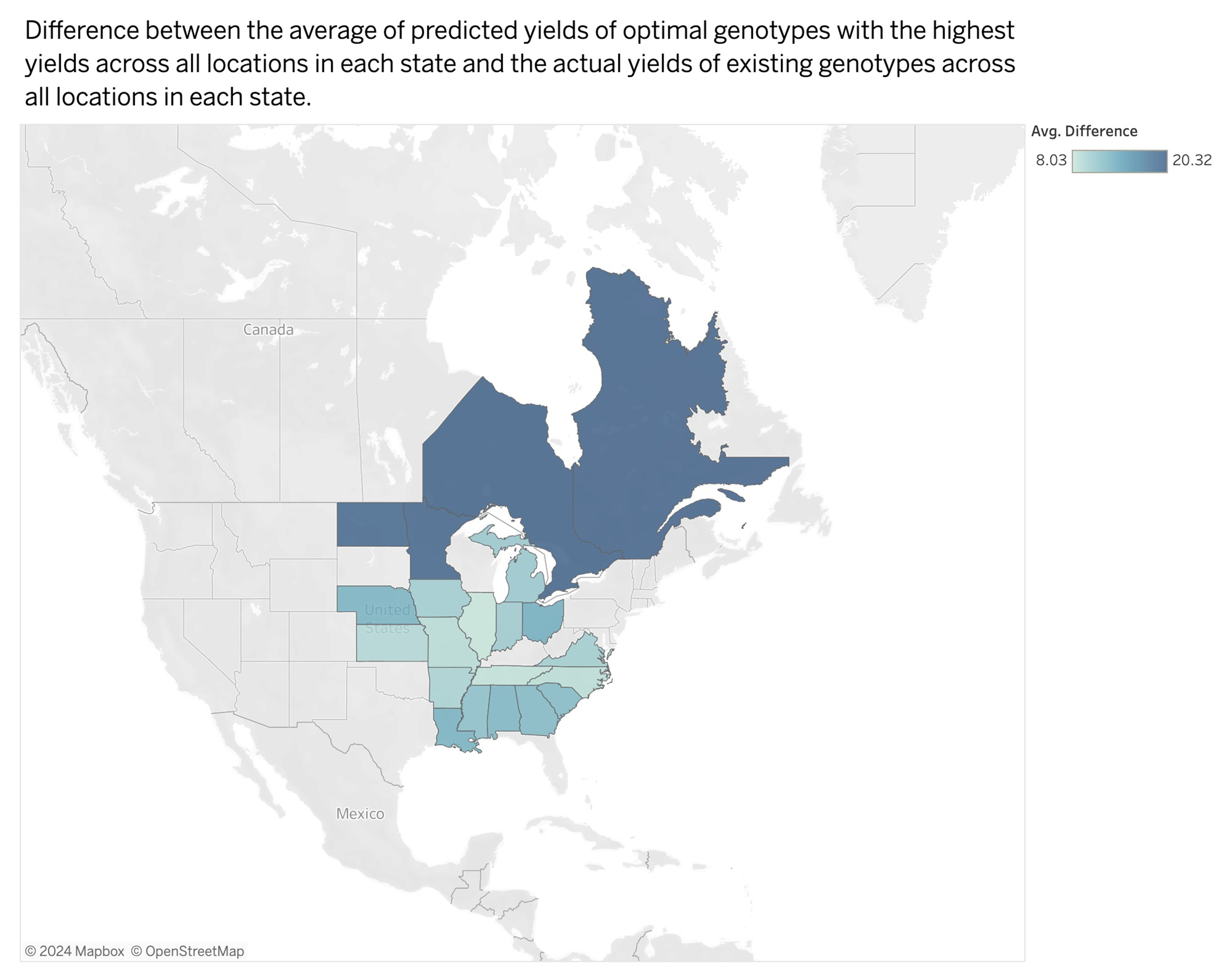

Supplement: Supplementary file 14 [file Image_12.jpeg]

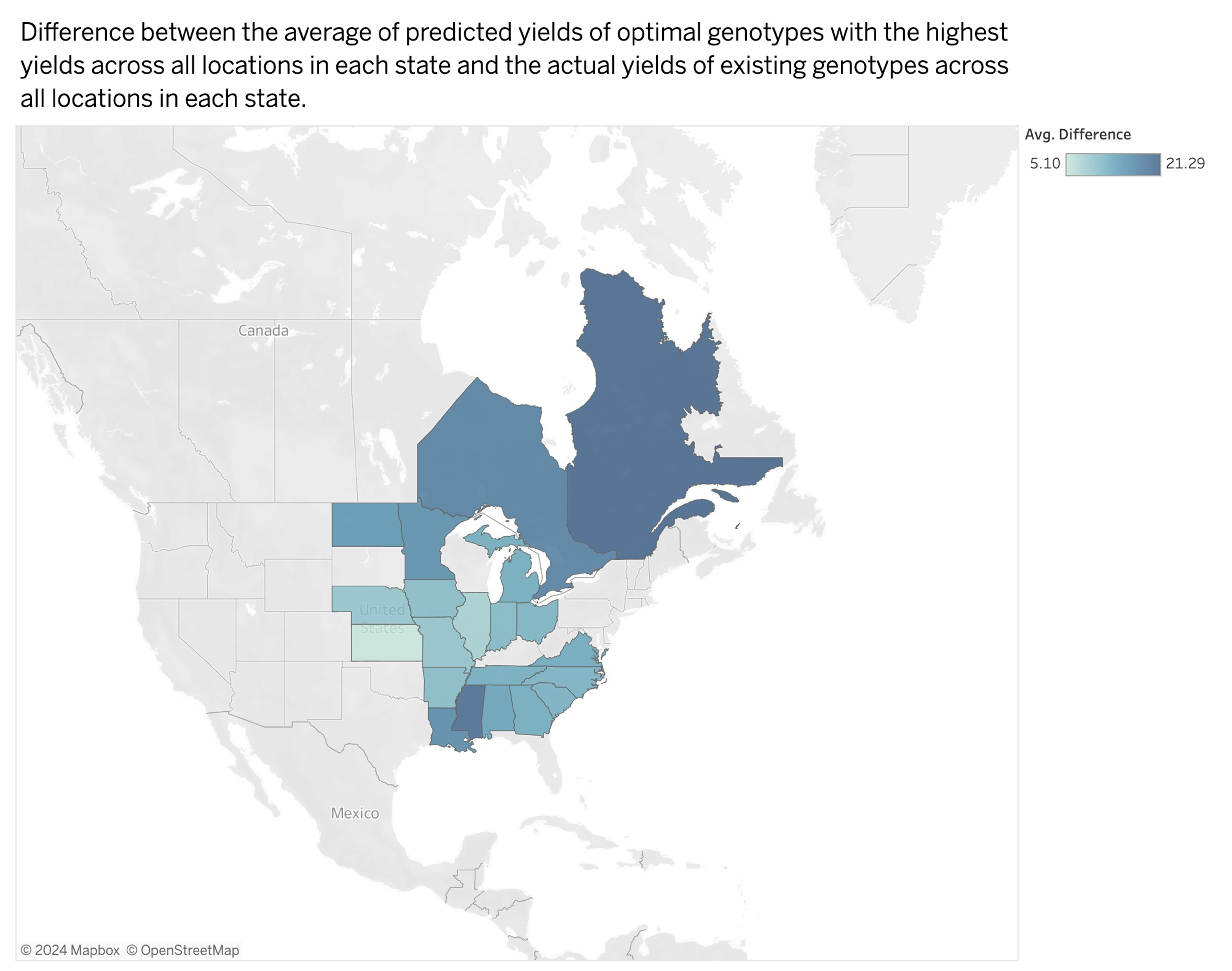

Supplement: Supplementary file 15 [file Image_13.jpeg]
